# Distinctive regulatory architectures of germline-active and somatic genes in *C. elegans*

**DOI:** 10.1101/gr.265934.120

**Published:** 2020-12

**Authors:** Jacques Serizay, Yan Dong, Jürgen Jänes, Michael Chesney, Chiara Cerrato, Julie Ahringer

**Affiliations:** The Gurdon Institute and Department of Genetics, University of Cambridge, Cambridge CB2 1QN, United Kingdom

## Abstract

RNA profiling has provided increasingly detailed knowledge of gene expression patterns, yet the different regulatory architectures that drive them are not well understood. To address this, we profiled and compared transcriptional and regulatory element activities across five tissues of *Caenorhabditis elegans*, covering ∼90% of cells. We find that the majority of promoters and enhancers have tissue-specific accessibility, and we discover regulatory grammars associated with ubiquitous, germline, and somatic tissue–specific gene expression patterns. In addition, we find that germline-active and soma-specific promoters have distinct features. Germline-active promoters have well-positioned +1 and −1 nucleosomes associated with a periodic 10-bp WW signal (W = A/T). Somatic tissue–specific promoters lack positioned nucleosomes and this signal, have wide nucleosome-depleted regions, and are more enriched for core promoter elements, which largely differ between tissues. We observe the 10-bp periodic WW signal at ubiquitous promoters in other animals, suggesting it is an ancient conserved signal. Our results show fundamental differences in regulatory architectures of germline and somatic tissue–specific genes, uncover regulatory rules for generating diverse gene expression patterns, and provide a tissue-specific resource for future studies.

Cell type–specific transcription regulation underlies production of the myriad of different cells generated during development. Regulatory elements (i.e., promoters and enhancers) are key sequences that direct appropriate spatiotemporal gene expression patterns, and they can have diverse activities, ranging from ubiquitous to highly cell type specific (Smith et al. 2007; Cusanovich et al. 2018; Andersson and Sandelin 2019; Liu et al. 2019). The composition, activity, and arrangement of regulatory elements define the regulatory grammar that controls patterns of gene transcription across development (Levine 2010; Ong and Corces 2011; Spitz and Furlong 2012; Heinz et al. 2015; Rajendra Sonawane et al. 2017), and mutation or perturbation of their spatial organization can lead to pathologies (Lupiáñez et al. 2016).

Previous studies have provided important and increasingly detailed knowledge of features of transcription regulation in eukaryotes. Different regulatory architectures have been observed, ranging from single promoters to complex structures involving multiple regulatory elements, which can operate redundantly, hierarchically, additively, or synergistically (Herr 1993; Davuluri et al. 2008; Guerrero et al. 2010; Whyte et al. 2013; Bahr et al. 2018; Osterwalder et al. 2018). Work on human cells suggests that housekeeping genes are primarily regulated by a single core promoter, whereas tissue-specific genes rely on additional regulatory elements (Ernst et al. 2011). Moreover, differences in sequence features, patterns of transcription initiation, and nucleosome arrangement characterize promoters with different activities (Lenhard et al. 2012; Haberle and Lenhard 2016). Yet, cell type–specific differences are still not well understood. More comprehensive genome-wide in vivo studies of regulatory grammar would directly address how specific gene expression patterns in different tissues are achieved and whether expression is governed by distinct regulatory architectures. *Caenorhabditis elegans* is a powerful system to study tissue-specific regulatory grammar, with its small genome, simple anatomy, and wealth of genomic data (The *C. elegans* Sequencing Consortium 1998; Gerstein et al. 2010; Ho et al. 2014; Jänes et al. 2018; Kudron et al. 2018). To investigate tissue-specific regulatory grammars, we profiled and compared nuclear transcriptomes and chromatin accessibility in sorted *C. elegans* tissues. Analyses of these rich data sets revealed shared and distinct features of ubiquitous and tissue-specific regulatory architectures.

## Results

### Tissue-specific profiling of chromatin accessibility and gene expression in *C. elegans* tissues

**Figure 1.**
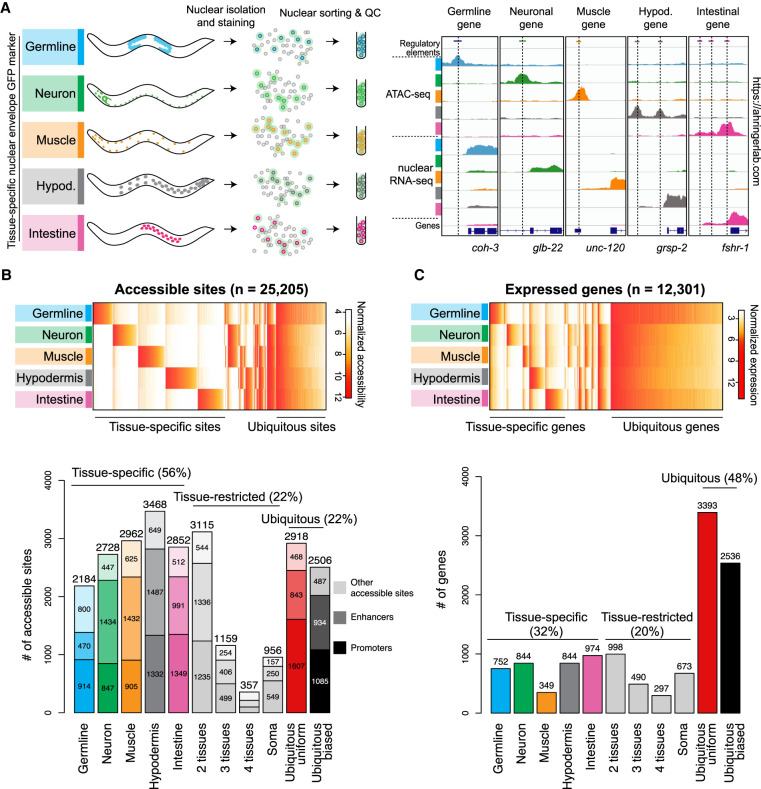
Tissue-specific profiling of chromatin accessibility and gene expression in *C. elegans* tissues. (*A*) Procedure to perform tissue-specific nuclear RNA-seq and ATAC-seq experiments. Representative results at known tissue-specific loci are shown on the *right*. (*B*, *top*) Heatmap of normalized accessibility (log_2_ RPM) for 25,205 classified sites. (*Bottom*) Classification of the accessible sites into tissue-specific, tissue-restricted, or ubiquitous classes. Protein-coding promoters are in dark colors, enhancers are lighter, and other accessible sites (e.g., noncoding promoters, unassigned promoters, other elements) are lightest. (*C*, *top*) Heatmap of normalized gene expression (log_2_ TPM) for 12,301 classified protein-coding genes. (*Bottom*) Classification of genes into tissue-specific, tissue-restricted, or ubiquitous classes. For classification procedure, see Methods. Unclassified sites and genes are not shown.

To investigate the regulatory chromatin of different cell types and how it relates to gene expression, we developed a procedure to isolate nuclei from individual tissues in *C. elegans* by expressing GFP tags on the outside of the nuclear envelope using tissue-specific promoters and by isolating labelled nuclei using fluorescent-activated nuclear sorting (see Methods) (Fig. 1A; Supplemental Figs. S1, S2). Here we focus on tissue-specific gene expression using transgenes active in the germline or the four major somatic tissues of *C. elegans* (muscle, hypodermis, intestine, and neurons). To isolate fully differentiated somatic tissues and avoid inclusion of nuclei from embryos, we synchronized animals at the L1 stage then fed them until the population predominantly contained late L4s and young adults without embryos. We obtained nuclei of high purity from each tissue (97.4% ± 1.27 SD) (Supplemental Table S1). The samples cover ∼90% of cells of adult worms but do not include the pharynx, glia, or somatic gonad.

We previously defined 42,245 accessible elements across *C. elegans* development and ageing in whole animals (Jänes et al. 2018). Because most genes in *C. elegans* are *trans*-spliced to a 22-nt leader RNA (Blumenthal 2012), leading to removal and degradation of the initial 5′ sequence between the promoter and the *trans*-splice site, the beginning of the mature mRNA does not usually correspond to the transcription initiation site. Therefore, nuclear RNA-seq patterns were used to annotate accessible sites identified using ATAC-seq (Jänes et al. 2018). Accessible elements were annotated as promoters based on the presence of nuclear transcription initiation signal within 125 bp downstream from the peak of accessibility and transcription elongation signal linking the element to a gene (unidirectional promoters) or genes (bidirectional promoters). Elements with transcription initiation signal but lacking transcription elongation signal were annotated as putative enhancers, and those lacking signals of transcriptional activity were classified as “other.”

To classify the tissue specificity of gene expression and accessibility and to identify new elements, we performed ATAC-seq and nuclear RNA-seq on sorted nuclei from the five tissues. Biological replicates were highly concordant (Supplemental Fig. S2D,E), and known tissue-specific loci showed expected activities (Fig. 1A). By using the procedures by Jänes et al. (2018), we identified and annotated 5269 additional accessible elements through these new data, bringing the total to 47,514. This added 2218 new putative enhancers (hereafter referred to as enhancers) and 901 new promoters; 11,806 protein-coding genes (58%) have at least one high-confidence promoter.

Finally, we classified the tissue specificity of element accessibility and gene expression using a set of conservative rules (see Methods). Excluding elements and genes with low signal in all assessed samples, 25,205 (53%) of the 47,514 accessible sites and 12,301 (61%) of the 20,222 protein-coding genes were classified (Fig. 1B,C; Supplemental Table S2).

We observed that the chromatin accessibility of regulatory elements is largely tissue specific. The majority of regulatory elements (56%) are accessible in only a single tissue, with the rest having tissue restricted (22%) or ubiquitous accessibility (22%); the latter were split into those with relatively uniform accessibility (less than threefold difference between any two tissues) and those with biased accessibility (Fig. 1B). For gene expression, the largest class of genes (48%) had ubiquitous expression, with the remainder having tissue-specific (32%) or tissue-restricted (20%) expression (Fig. 1C). The gene expression classification showed good overlap with previously published annotations (Supplemental Fig S2G,H; Cao et al. 2017; Kaletsky et al. 2018). We observed that the nuclear RNA data sets have minor cross-contamination that appears, in part, to be bulk cytoplasmic RNA released during nuclear isolation, resulting in tissue-specific genes with high expression (e.g., muscle myosin gene *unc-54*) being classified as ubiquitous biased. Hereafter, when studying ubiquitous genes and elements, we specifically focus on the ubiquitous-uniform class and for simplicity refer to them as “ubiquitous.”

The data provide a comprehensive view of chromatin accessibility and transcriptional landscapes in the five major *C. elegans* tissues. To facilitate access and analyses of these new tissue-specific and previous development data sets (Jänes et al. 2018), we created RegAtlas, a *C. elegans* regulatory atlas (https://ahringerlab.com/). Below, we analyze features of genes and regulatory elements active in different tissues.

### Germline-active and soma-restricted genes have distinctive regulatory architectures

**Figure 2.**
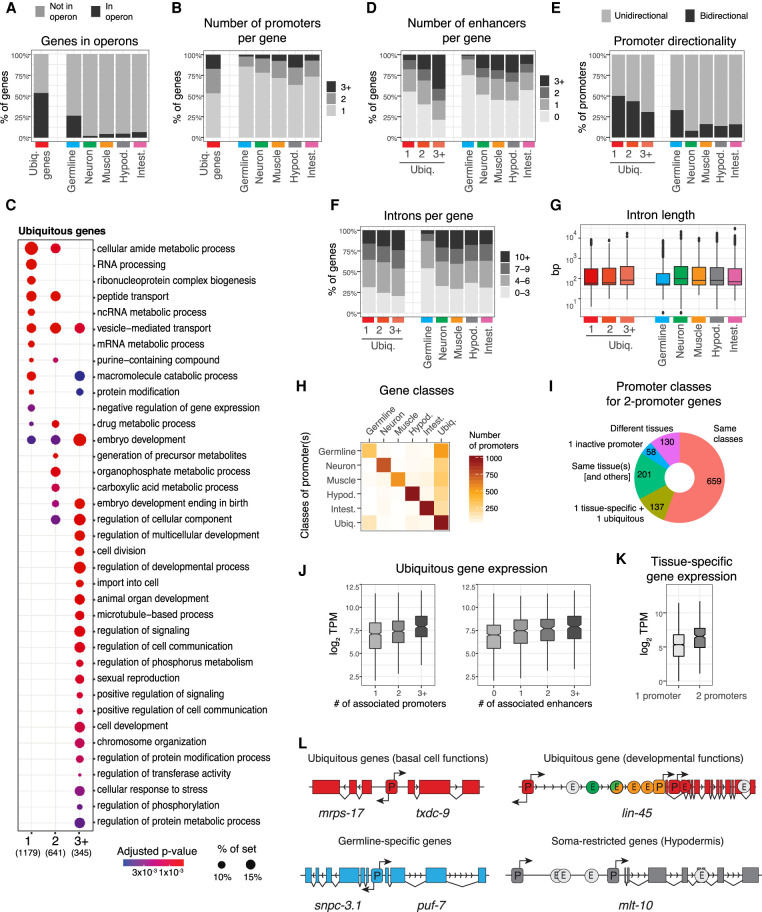
Regulatory architectures of ubiquitous, germline, and soma-restricted genes have distinctive features. (*A*) Percentage of genes organized in an operon for each gene class. (*B*) Percentage of genes with one, two, or three or more promoters for each gene class. (*C*) GO terms from Biological Process ontology enriched in ubiquitous genes with one, two, or three or more annotated promoters. (*D*) Percentage of genes with zero, one, two, or three or more enhancers associated with genes of each expression class. Only genes with at least one annotated promoter are considered. (*E*) Percentage of unidirectional or bidirectional protein-coding promoters for each gene class. (*F*) Percentage of genes with the indicated number of introns for each gene class. (*G*) Intron length for each gene class. (*H*) Classes of promoters associated with genes of each expression class. Only the major promoter classes are displayed. For all results, see Supplemental Table S2. (*I*) Concordance of promoter classes for genes with two promoters. (*J*) Gene expression levels in whole young adults for ubiquitous genes with one, two, or three or more promoters (*left*) or with zero, one, two, or three or more enhancers (*right*). (*K*) Gene expression levels of tissue-specific genes with one promoter or two promoters specifically active in the same tissue. In panels *B* through *K*, only first genes in operons and nonoperon genes were considered. (*L*, *left*) Examples of the simple regulatory architecture shared by ubiquitous genes and germline-specific genes. (*Right*) Examples of more complex architectures found at developmental ubiquitous genes (e.g., *lin-45*) or somatic tissue–specific genes (e.g., *mlt-10*).

To investigate whether general rules could be discerned that govern different types of spatial expression patterns, we focused on genes with ubiquitous or tissue-specific expression and compared the number, type, and arrangement of regulatory elements associated with genes from each class. About 15% of *C. elegans* genes are organized in operons (Reinke and Cutter 2009), where two or more genes are initially transcribed into a single transcript that is separated by *trans*-splicing. Therefore, for analyses involving promoters, we only included nonoperon genes and first genes in operons. As reported previously (Reinke and Cutter 2009), we found that genes organized in operons preferentially have ubiquitous or germline-specific patterns of expression (Fig. 2A).

As expected, most (77%) ubiquitously expressed genes with at least one classified promoter are associated with a ubiquitously active promoter (Supplemental Table S2). We observed that half (54%) of the ubiquitous genes have just a single promoter, whereas 16% have a relatively complex regulatory architecture containing three or more promoters (Fig. 2B). To explore these differences, we separated ubiquitously expressed genes into groups based on promoter number (one, two, or three or more). We observed that single-promoter genes are enriched for functions in basic cellular processes, whereas those with three or more promoters are enriched for developmental functions (Fig. 2C). Multipromoter ubiquitous genes also have more enhancers than single-promoter genes, are more often controlled by unidirectional promoters, and have more and longer introns (Fig. 2D–G).

As for ubiquitous genes, tissue-specific genes are generally associated with one or more promoters specific for the corresponding tissue (78%) (Fig. 2H). However, we observed that a group of genes with germline-specific expression have ubiquitously accessible promoters (Fig. 2H). We found that these genes are enriched for being targets of the repressive Rb/DREAM complex (13-fold enrichment, *P*-value = 5.10^−13^), and in line with this, they are enriched for cell cycle and cell division functions (Supplemental Fig S3A,B; Latorre et al. 2015; Goetsch et al. 2017). This suggests that the predominantly germline expression of these genes is achieved via their silencing in somatic tissues (Petrella et al. 2011; Wu et al. 2012).

By comparing the different tissue-specific classes, we found that germline-specific genes show extensive differences compared with somatic genes. First, germline-specific genes have fewer promoters and enhancers than somatic genes (Fig. 2B,D); 65% of germline-specific genes with at least one classified promoter have a single promoter and no associated enhancer compared with 38% of somatic genes. The promoters of germline genes are more often bidirectional than those of somatic genes (Fig. 2E), and germline genes also have fewer and shorter introns, similar to ubiquitously expressed single-promoter genes (Fig. 2F,G).

A significant fraction of expressed genes with at least one annotated promoter (33%) have more than one promoter, and alternative promoters are frequently active in the same tissue (Fig. 2I; Supplemental Fig S3C; Supplemental Table S2), suggesting that alternative promoters may play a role in the regulation of expression levels. To investigate this, we examined the relationship between the number of regulatory elements and gene expression level. Among ubiquitously expressed genes, we found that the number of promoters and enhancers was positively correlated with gene expression (Fig. 2J). Similarly, tissue-specific genes with two tissue-specific promoters have higher gene expression levels than those with only one (Fig. 2K). We also note that 15% of the ubiquitously expressed genes with two promoters have one tissue-specific promoter in addition to a ubiquitously active one, which could be a mechanism to increase gene expression specifically in a particular tissue (Supplemental Fig. S3D). These results suggest that an important but often overlooked role of regulatory elements is to augment gene expression rather than being necessary for its expression per se. This could explain some cases in which deletion of an individual regulatory element does not have an obvious effect on gene expression, despite the regulatory element having transcriptional activity in transgenic assays (Dukler et al. 2017; Catarino and Stark 2018).

To summarize, we found that the regulatory architecture of genes is related to their function and expression pattern (Fig. 2L). Ubiquitous genes required for fundamental cellular processes and germline-specific genes tend to have a simple architecture consisting of a single promoter that is often bidirectional. In contrast, ubiquitous genes with functions associated with multicellular life often have a more complex architecture of multiple regulatory elements that can have diverse tissue specificity. Somatic tissue–specific genes usually have one or more regulatory elements accessible only in the matching tissue. Finally, the positive relationship between gene expression and the number of regulatory elements supports a role in modulating the level of gene expression.

### Ubiquitous and germline-specific promoters have a stereotypical architecture with well-positioned nucleosomes

The tissue-specific differences in gene regulatory architectures prompted us to investigate whether differences also occurred at the level of promoters. By comparing accessibility patterns of different classes of promoters, we observed that germline-specific and ubiquitously active promoters were flanked by regions of increased accessibility and associated with more nucleosome-sized ATAC-seq fragments, suggesting the presence of well-positioned nucleosomes (Supplemental Fig. S4A,B). The flanking ATAC-seq signal at germline promoters was also present in proliferative stages (L1 and L3 larval stages), indicating that it is not simply a characteristic of germline nuclei undergoing meiosis (Supplemental Fig. S4C).

**Figure 3.**
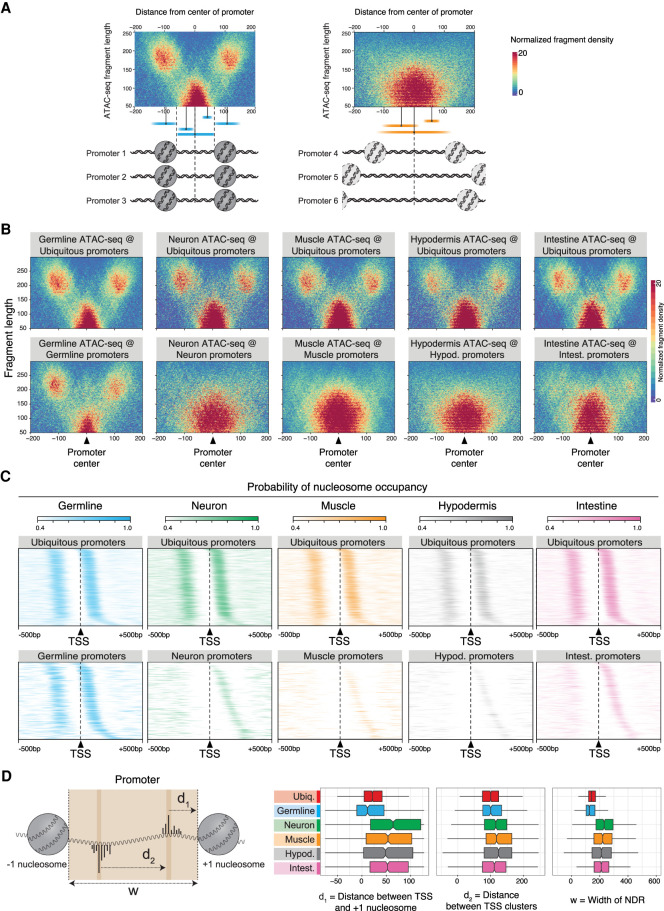
Ubiquitous and germline-specific promoters have a stereotypical architecture with well-positioned nucleosomes. (*A*) Interpretation of two ATAC-seq fragment density plots (also known as “V-plots”). The dense cluster of short fragments at the promoter centers represents the nucleosome-depleted region (NDR), whereas the dense clusters of longer fragments located −100 and +100 bp away from the promoter centers are indicative of aligned −1/+1 flanking nucleosomes. (*B*) ATAC-seq fragment density plots (V-plots) over different classes of promoters. The *x*-axis represents the distance between the fragment midpoint and the promoter center. The *y*-axis represents ATAC-seq fragment length. The color scale indicates the normalized density of ATAC-seq fragments. (*C*) Tissue-specific nucleosome occupancy probability over different classes of promoters aligned at their TSS. Only promoters with experimentally defined forward and reverse TSSs are considered. Rows are ordered by the distance between TSS and +1 nucleosome. (*D*, *left*) Schematic of the distance metrics measured in promoters: (d_1_) distance between the mode TSS and the +1 nucleosome edge; (d_2_) distance between modes of divergent TSSs within the same promoter; (w) width of the NDR. (*Right*) d_1_, d_2_, and w distance metrics for different classes of promoters. The metrics for ubiquitous promoters were measured using nucleosome occupancy probability track derived from whole young adult ATAC-seq data (Jänes et al. 2018).

To investigate this potential signature of positioned nucleosomes, we used ATAC-seq fragment density plots (also known as “V-plots”) (Henikoff et al. 2011) to visualize the distribution of fragment lengths relative to the distance to the promoter center. Over promoters flanked by positioned −1 and +1 nucleosomes, V-plots show stereotypical patterns with a central concentration of small fragments at the nucleosome-depleted region (NDR) and larger fragments over +1/−1 nucleosomes on either side of the NDR (Fig. 3A; Henikoff et al. 2011). In line with this, a signature of −1 and +1 nucleosomes is readily apparent at ubiquitous promoters in all tissues, as well as at germline-specific promoters (Fig. 3B). However, somatic tissue–specific promoters lack this signature of well-positioned +1/−1 nucleosomes (Fig. 3B; Supplemental Fig. S5A,B), regardless of the level of expression of the associated gene (Supplemental Fig. S5C) or promoter directionality (Supplemental Fig. S5D,E).

To explore this further, we used the ATAC-seq data to compute nucleosome occupancy probability profiles as previously described (Schep et al. 2015). This revealed a high probability of +1 and −1 nucleosome occupancy at consistent positions relative to transcription start sites (TSSs) of ubiquitous and germline-specific promoters (Fig. 3C; Supplemental Fig. S4D). In contrast, somatic tissue–specific promoters were characterized by lower −1 and +1 nucleosome occupancy and a larger range of nucleosome positions relative to TSSs (Fig. 3C; Supplemental Fig. S4D). We found that the 5′ edges of +1 nucleosomes at ubiquitous and germline-specific promoters have narrow distributions relative to TSSs, with median distances of 22 bp for ubiquitous promoters and 12 bp for germline-specific promoters. In contrast, +1 nucleosomes at somatic tissue–specific promoters have much wider distributions and larger median distances (Fig. 3C,D). We also observed that NDR widths are smaller, and divergent promoter TSSs are closer together for ubiquitous and germline-specific promoters compared with somatic tissue–specific promoters (Fig. 3D; Supplemental Fig. S4D). Of note, the median NDR widths at ubiquitous and germline-specific promoters are 140 bp and 125 bp, which would be too short to accommodate a nucleosome.

**Figure 4.**
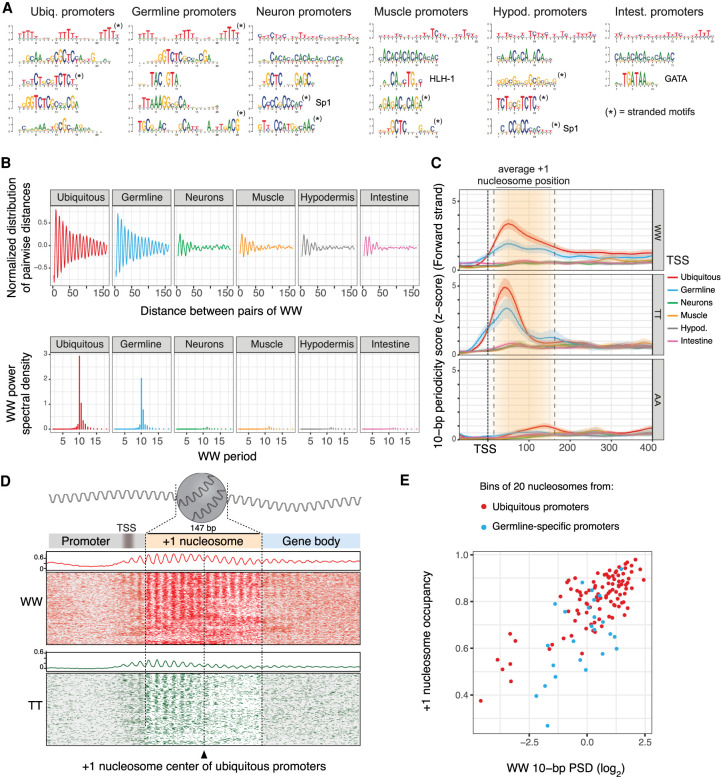
Ubiquitous and germline-specific promoters have strong 10-bp WW periodicity correlated with nucleosomes. (*A*) Motifs enriched in different classes of promoters. Sequences from −75 to +105 bp around the promoter centers were considered. (*B*, *top*) Normalized distribution of pairwise distances between WW dinucleotides found in the sequences from −50 bp to +300 bp relative to TSSs, for different classes of promoters. (*Bottom*) Associated WW power spectral densities (PSDs). (*C*) Metaplots of WW, TT, and AA 10-bp periodicity scores at different classes of promoters, aligned at TSSs. The +1 nucleosome position observed at ubiquitous and germline promoters (∼20–167 bp downstream from the TSS) is displayed by the shaded orange area delimited by dotted lines. (*D*) WW (red) and TT (green) dinucleotide occurrences observed at +1 nucleosomes of ubiquitous promoters (400-bp window centered at nucleosome dyads). Rows were shifted up to 5 bp to highlight the phased 10-bp periodic patterns. Summed dinucleotide occurrences are represented on *top* of each heatmap by a line plot. The average TSS positions of ubiquitous promoters (∼20 bp upstream of the +1 nucleosome edge) are displayed by the shaded gray area. (*E*) Correlation between +1 nucleosome occupancy and 10-bp WW periodicity in ubiquitous and germline-specific promoters. The +1 nucleosomes were binned by their nucleosome occupancy score, and the overall 10-bp WW periodicity was assessed in each bin (approximately 20 200-bp long nucleosomal sequences centered at nucleosome dyads). The *y*-axis represents the average nucleosome occupancy in each bin.

To identify sequence features that may be responsible for these differences, we performed motif analyses. We observed that ubiquitous and germline-specific promoters share a T-rich motif with 10-bp spacing that was not present at somatic tissue–specific promoters (Fig. 4A). Previous studies mostly performed in vitro or in yeast have implicated 10-bp WW (W = A/T) periodicity in nucleosome positioning and observed SS periodicity in antiphase with WW (Satchwell et al. 1986; Ioshikhes et al. 1996; Wang and Widom 2005; Johnson et al. 2006; Segal et al. 2006; Field et al. 2008; Mavrich et al. 2008a; Struhl and Segal 2013).

To investigate whether the T-rich motif we identified was part of a larger WW periodic signal involved in +1 nucleosome positioning at ubiquitous and germline-specific promoters in *C. elegans*, we measured WW dinucleotide periodicity from −50 bp to +300 bp relative to TSSs. We observed that ubiquitous and germline-specific promoter regions harbor a strong 10-bp periodic WW signal that extends for >150 bp and that the periodicity signal coincides with +1 nucleosome position (Fig. 4B,D; Supplemental Fig. S6A). Furthermore, at these promoters we found that 10-bp WW periodicity strength is correlated with +1 nucleosome occupancy (Fig. 4E). In contrast, the 10-bp periodic WW signal was not detected at somatic tissue–specific promoters, in line with the absence of positioned +1 nucleosomes at these promoters (Fig. 4B,C; Supplemental Fig. S6A,B). Therefore, an extended 10-bp periodic WW signal specific to ubiquitously active and germline active promoters is associated with nucleosome position and occupancy.

By examining the contribution of different dinucleotides to the WW signal, we found that TT periodicity peaks in the 5′ region of the +1 nucleosome of ubiquitous and germline-specific promoters, ∼50 bp downstream from the TSS, and makes a larger contribution than other dinucleotides (Fig. 4C,D; Supplemental Fig. S6A). A weaker AA periodic signal peaks at the 3′ edge of the nucleosome (Fig. 4C,D; Supplemental Fig. S6A), and AT and TA dinucleotides do not show any robust periodic signal (Supplemental Fig. S6A). We note that a 10-bp SS periodicity antiphase with WW is also present at ubiquitous and germline-specific promoters (Supplemental Fig. S6A,B).

The strength of the 10-bp periodic WW signal is similar at +1 nucleosomes of bidirectional and unidirectional ubiquitous promoters (Supplemental Fig. S6C). Periodicity is also present at −1 nucleosomes of the unidirectional promoters, although the signal is weaker (Supplemental Fig. S6C). We also note that WW periodicity strength differs among ubiquitous promoters of ubiquitous genes. WW periodicity is stronger at single-promoter genes, which are enriched for basal cell functions, compared with ubiquitous promoters of genes with three or more promoters, which are enriched for developmental functions (Fig. 2C; Supplemental Fig. S6C).

We next investigated the tissue specificity and position of other promoter elements. The Inr initiator sequence, the Sp1 motif, and the TATA-box are three well-known core promoter elements that have been previously observed in *C. elegans* promoters (Saito et al. 2013; Chen et al. 2013). Inr motifs were detected in all promoter classes; however, somatic tissue–specific promoters showed higher enrichment than ubiquitous and germline-specific promoters (Supplemental Fig. S7; Supplemental Table S3). We further observed that the Sp1 and TATA box motifs were both predominantly associated with somatic tissue–specific promoters, with large and unexpected tissue biases (Supplemental Fig. S7; Supplemental Table S3). The Sp1 motif, peaking at −45 bp from the TSS, is enriched at neural, muscle, and hypodermal promoters but not at intestinal promoters, whereas the TATA-box motif was predominantly found at hypodermal and intestinal promoters. We also observed that somatic tissue–specific promoters share repeated dinucleotide composition biases not found in ubiquitous or germline-specific promoters (Fig. 4A; Supplemental Fig. S7; Supplemental Table S3).

The de novo motif analyses also uncovered motifs associated with promoters active in single tissues (Fig. 4A; Supplemental Fig. S7; Supplemental Table S3). For example, as expected, many intestinal promoters harbor a GATA motif, whereas the HLH-1 motif is found specifically at muscle promoters (Supplemental Fig. S7; Chen et al. 1994; McGhee et al. 2007). These motifs and others have peak positions within the NDR, often ∼45 bp upstream of the TSS (Supplemental Fig. S7). Thus, there are tissue-specific differences in both core promoter elements and TF binding motifs.

In summary, our results uncover two largely different types of promoter architecture. Ubiquitous and germline-specific promoters have well-positioned +1/−1 nucleosomes that are highly associated with a periodic 10-bp WW signal and stereotypically positioned with the 5′ edge ∼20 bp downstream from the TSS, and they have relatively short NDRs. In contrast, +1 nucleosomes of somatic tissue–specific promoters have low occupancy and inconsistent positioning relative to TSSs, and NDRs are wider. In addition, core promoter and transcription factor motifs show strong tissue biases.

### Ten-base-pair WW periodicity at ubiquitous promoters is a feature of nonmammalian genomes

We next asked whether a 10-bp periodic WW signal is a feature associated with +1 nucleosomes of ubiquitous promoters of other animals. Ten-base-pair periodic WW sequences have been observed at +1 nucleosomes in yeast, *Drosophila*, and zebrafish but not in mammals (Albert et al. 2007; Mavrich et al. 2008a,b; Tolstorukov et al. 2009; Ioshikhes et al. 2011; Haberle et al. 2014; Wright and Cui 2019). However, whether 10-bp WW periodicity is associated with promoters of particular types has not been investigated.

**Figure 5.**
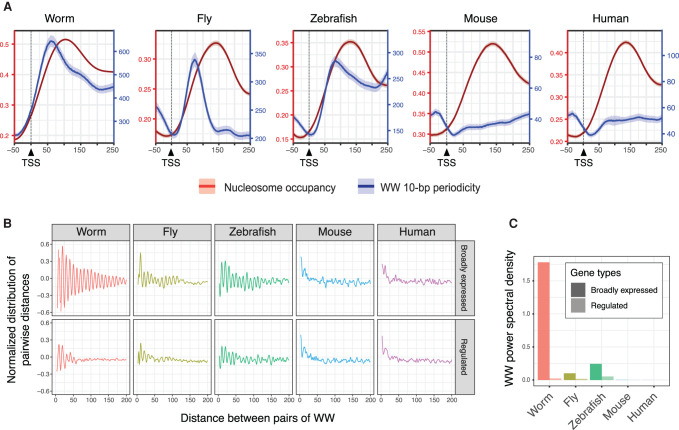
Ten-base-pair WW periodicity at ubiquitous promoters is a feature of nonmammalian genomes. (*A*) Nucleosome occupancy probability scores (red; *left* axis) and 10-bp WW periodicity (blue; *right* axis) at worm, fly, zebrafish, mouse, and human TSSs. (*B*) Normalized distribution of pairwise distances between WW dinucleotides found in the sequences from −50 bp to +300 bp relative to TSSs, for genes with broad expression (*top* row; 20% lowest gene expression CV scores) or regulated expression (*bottom* row; 20% highest gene expression CV scores) in worm, fly, zebrafish, mice, and human. (*C*) Associated WW power spectral density values at a 10-bp period.

We first examined TSS sets that represent all genes in *Drosophila*, zebrafish, mouse, and human (see Methods). As expected, we detected 10-bp WW periodicity signals downstream from *Drosophila* and zebrafish TSSs, but not human TSSs, and we found that this signal was also not detected in mouse (Fig. 5A). As in *C. elegans*, we observed that the WW periodicity signals in *Drosophila* and zebrafish peaked in the 5′ half of +1 nucleosomes (Fig. 5A).

We then investigated subsets of promoters to ask whether 10-bp WW periodicity signals are associated with ubiquitously active promoters, and to compare with signals at promoters with regulated activity. By using the coefficient of variation of gene expression (CV) as a metric, we considered genes in the bottom 20% of CV values to have broad ubiquitous expression and those in the top 20% to have highly regulated expression (e.g., tissue specificity). As found in *C. elegans*, we observed that promoters of broadly expressed genes in *Drosophila* and zebrafish have higher 10-bp WW periodicity signals than those of highly regulated genes. In contrast, neither the broadly active nor the regulated groups of mouse and human promoters had detectable WW periodicity signals (Fig. 5B,C). These results suggest that 10-bp WW periodicity signals are a conserved feature of ubiquitously active promoters in nonmammalian animals.

## Discussion

Determining the regulatory architectures that drive different gene expression patterns is necessary for understanding how the genome encodes development. Through comprehensive analyses of gene expression and chromatin accessibility in five *C. elegans* tissues covering ∼90% of cells, we show that most genes have either ubiquitous or tissue-specific expression, and we describe extensive differences between their regulatory architectures. The expression of ubiquitous genes involved in basic biological processes as well as that of germline-specific genes is often controlled by single promoters, whereas soma-specific and ubiquitous genes involved in developmental processes have more alternative promoters and enhancers.

We also found that the majority of regulatory elements have tissue-specific accessibility, and we identified differences in sequence composition between promoters active in different tissues. We found that a strong +1 nucleosome position coinciding with a 10-bp periodic WW signal is a key feature of ubiquitous and germline-specific promoters in *C. elegans*. The association of 10-bp WW periodicity and nucleosome rotational position was first noted by Travers and colleagues in chicken and is thought to aid nucleosome positioning by conferring sequence-dependent bendability to the DNA polymer (Zhurkin et al. 1979; Trifonov 1980; Drew and Travers 1985). Such periodicity has been observed in nucleosomal sequences in different eukaryotes, including *C. elegans*, but its specific association with different gene types was unknown (Satchwell et al. 1986; Ioshikhes et al. 1996, 2011; Widom 2001; Johnson et al. 2006; Segal et al. 2006; Peckham et al. 2007; Field et al. 2008; Mavrich et al. 2008a,b; Struhl and Segal 2013; Forrest et al. 2014; Haberle et al. 2014; Dreos et al. 2016; Pich et al. 2018). We note that 10-bp periodic An/Tn-clusters (PATCs) have also been shown to be associated with introns of *C. elegans* germline expressed genes located in repressive chromatin domains and to help facilitate their transcription (Fire et al. 2006; Frøkjær-Jensen et al. 2016). The mechanism of this regulation is unclear, but the 10-bp periodic A/T signals suggest that nucleosome positioning may play a role.

In contrast to ubiquitous and germline-specific promoters, +1 nucleosomes of somatic tissue–specific promoters are not associated with a 10-bp WW periodicity signal, have lower occupancy, and have inconsistent position relative to the TSS. Instead, we observed strong biases in the enrichment of core motifs at these promoters. TATA boxes are primarily found in hypodermal and intestinal promoters, whereas Sp1 motifs are most highly enriched in neuronal promoters. In addition, tissue-specific motifs are present, and these often have peak positions around −50 bp relative to the mode TSS.

**Figure 6.**
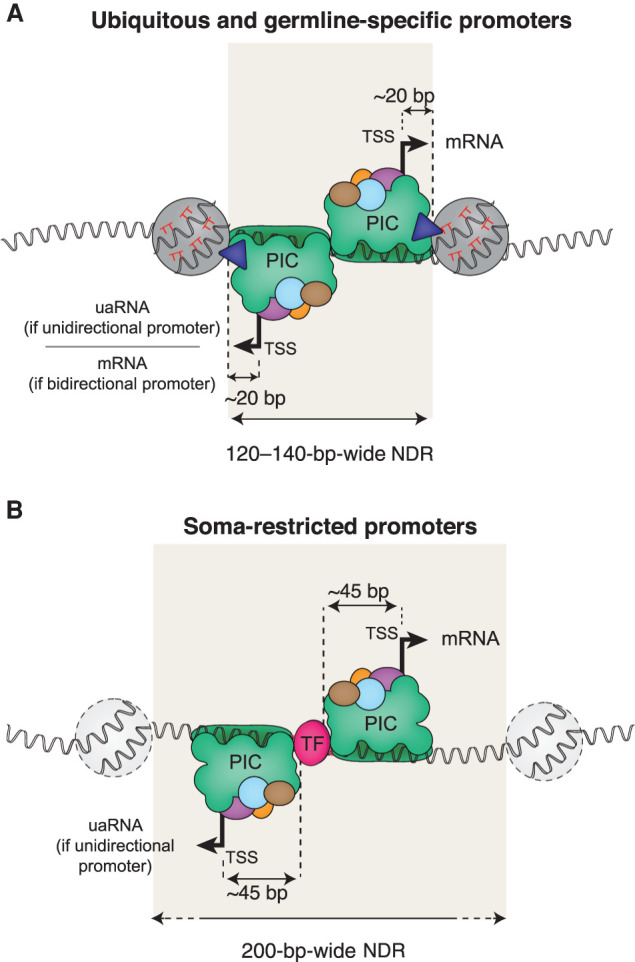
Two models of preinitiation complex (PIC) positioning at promoters. The nucleosome organization and sequences features found in ubiquitous, germline-specific, and somatic tissue–specific promoters suggest that two models of PIC recruitment exist. (*A*) In ubiquitous and germline-specific promoters (i.e., germline-active promoters), nucleosomes flank a narrow 120- to 140-bp-wide NDR. Positioning of these nucleosomes is facilitated by the underlying DNA sequence, which harbors highly periodic WW (mainly TT) dinucleotides. Thus, the PIC assembling at the NDR is physically constrained by the +1 nucleosome edge, resulting in transcription initiation ∼20 bp upstream of the +1 nucleosome edge. Many of these promoters lead to bidirectional elongative transcription. Otherwise, upstream-antisense RNA (uaRNA) are transcribed. (*B*) In soma-restricted promoters, NDRs are wider (>200 bp), and flanking nucleosomes are weakly positioned and not reproducibly aligned relative to the TSS. Core and transcription factors recruited to the NDR facilitate assembly and positioning of the PIC, resulting in transcription initiation −45 to −50 bp downstream.

Structural studies of the preinitiation complex (PIC) showed that it covers the region from about −45 bp to +20 bp relative to the TSS (Louder et al. 2016; Robinson et al. 2016; Schilbach et al. 2017). The 5′ edge of +1 nucleosomes at *C. elegans* ubiquitous and germline promoters are located ∼ 20 bp downstream from the TSS, which would be at the 3′ edge of the PIC. This supports the model initially proposed in yeast whereby a positioned +1 nucleosome could facilitate PIC complex assembly by interacting with TFIID (Fig. 6A,B; Jiang and Pugh 2009). At soma-specific promoters, which lack strongly positioned nucleosomes, the binding of core or tissue-specific TFs ∼ 45 bp upstream of the TSS might help to locally recruit and/or to position the PIC (Fig. 6A,B). These models are not mutually exclusive, and additional mechanisms also contribute to promoter activity.

Similar to *C. elegans*, we observed that a 10-bp WW periodicity signal is also associated with promoter +1 nucleosomes of broadly expressed genes in zebrafish and *Drosophila*. This is consistent with a previously described enrichment of 10-bp periodicity in AA and TT dinucleotides downstream from zygotic TSSs in zebrafish (Haberle et al. 2014). A weak genome-wide AA/TT periodicity was previously noted in *Drosophila* but not associated with any gene feature (Mavrich et al. 2008b). In contrast, the periodic WW signal is not detected at promoters of broadly expressed genes in mouse and human, despite their having well-positioned +1 nucleosomes. This is consistent with reports showing a low 10-bp WW periodicity in mammal genomes, either around TSSs (Tolstorukov et al. 2009; Wright and Cui 2019) or genome-wide (Pich et al. 2018). Multiple factors have been shown to contribute to nucleosome positioning in eukaryotes, including intrinsic DNA sequence, chromatin remodelers, DNA-binding proteins, and RNA polymerase machinery (Jiang and Pugh 2009; Struhl and Segal 2013). We suggest that 10-bp WW periodicity is an ancient conserved signal that contributes to +1 nucleosome positioning at ubiquitously active promoters of nonmammalian eukaryotes, especially those of genes with basal cell functions, whereas nucleosome positioning in mammals may rely on other mechanisms (Struhl and Segal 2013).

In addition to illuminating understanding of regulatory architectures, we provide extensive data sets and annotation of gene expression and accessible chromatin across tissues, available at the *C. elegans* regulatory atlas (RegAtlas) (Supplemental Fig. S8; https://ahringerlab.com/RegAtlas/). These data and tools will be key resources that facilitate future studies of *C. elegans* gene expression regulation by the scientific community.

## Methods

### Nuclear sorting

Animals were obtained by growing synchronized starved L1 larvae at 25 C in standard S-basal medium with HB101 bacteria for 40–42 h, resulting in collections predominantly containing late L4s and young adults with no embryos. After sucrose flotation and washing in M9 buffer, worms were frozen into “popcorn” by dripping concentrated slurry into liquid nitrogen. Nuclei were isolated as previously detailed (Jänes et al. 2018), with minor modifications. Approximately 20,000 to 200,000 frozen worms were broken by smashing using a BioPulverizer, and then the frozen powder was thawed in 8 mL egg buffer (25 mM HEPES at pH 7.3, 118 mM NaCl, 48 mM KCl, 2 mM CaCl_2_, 2 mM MgCl_2_). Broken worms were pelleted by spinning at 800*g* for 3 min and then resuspended in 8 mL of buffer A (0.3 M sucrose, 10 mM Tris at pH 7.5, 10 mM MgCl_2_, 1 mM DTT, 0.5 mM spermidine, 0.15 mM spermine, protease inhibitors [Roche complete, EDTA free], and 0.025% IGEPAL CA-630). The sample was dounced (two strokes) in a 14-mL stainless steel tissue grinder (VWR) and then spun at 100*g* for 6 min to pellet remaining worm fragments. The supernatant was kept (nuclei batch 1) and the pellet resuspended in a further 7 mL of buffer A and dounced for 30 strokes. This was spun at 100*g* for 6 min to pellet debris, and the supernatant was kept (nuclei batch 2). The first fraction was enriched for germline nuclei, whereas the second fraction was enriched for somatic nuclei. Nuclei quality was assessed by microscopy.

Following isolation, nuclei were immunostained by adding phycoerythrin-coupled anti-GFP antibody (Biolegend 338003) at 1:200 in 7 mL of buffer A, and 280 units of murine RNase inhibitor (M0314S) were added to protect RNA from being degraded. Nuclei were kept slowly rotating at 4°C in the dark for 1–16 h. Debris was removed by spinning at 100*g* for 6 min at 4°C, and then nuclei were pelleted (2000*g* for 20 min at 4°C), washed in 6 mL of buffer A, and resuspended in buffer A containing 80 U/mL murine RNase inhibitor at a concentration of approximately 10–15 million nuclei per milliliter. Finally, nuclei were filtered on a 30-µm mesh (CellTrics 04-0042-2316) and stained with 0.025 µg/mL DAPI. Nuclei quality was assessed immediately before sorting by microscopy.

Nuclear sorting was performed at 4°C using a Sony SH800Z sorter fitted with a 100-µm sorting chip and auto-calibrated. Nuclei were gated using the DAPI signal, and PE-positive nuclei were gated using PE-H/BSC-A signal. DAPI gating depended on which nuclei were being sorted (e.g., intestine nuclei are 32N). A recording speed of more than 15,000 nuclei per second ensured a sorting efficiency >80%. Nuclei were sorted into 15-mL Falcon tubes containing 500 µL of buffer A with 800U/mL murine RNase inhibitor. Nuclei were sorted in batches of 1 million and then processed for downstream applications. The purity and integrity of each batch of nuclei were assessed by recording an aliquot of sorted nuclei in a second pass in the sorter and by microscopy. All sorted samples used in this study had a purity >95%. Sorted nuclei were intact, as revealed by the circular DAPI signal observed, as well as the GFP signal outlining the nuclear envelope (see Supplemental Fig. S2B).

### ATAC-seq

One million sorted nuclei were pelleted (2000*g* for 20 min at 4°C) and resuspended in 1× Tn5 buffer (10 mM Tris at pH 8, 5 mM MgCl_2_, 10% DMF) at a final concentration of about 500,000 nuclei per milliliter; 2.5 µL of Tn5 (Illumina FC-121-1030) was added to 47.5 µL (about 25,000 nuclei) of the suspension. ATAC-seq was then performed as previously described (Jänes et al. 2018). ATAC-seq libraries were generated from two biological replicates for each tissue and were sequenced in both single-end and paired-end modes. Single ATAC-seq libraries were made for L1 and L3 muscle (SE-sequenced) and L3 germline (PE-sequenced). PGC-specific ATAC-seq data at the L1 stage were obtained from Lee et al. (2017).

### RNA-seq

RNA was extracted from 1 million sorted and washed nuclei using the standard procedure (Jänes et al. 2018). A minimum of 20 ng of total nuclear RNA was used to make long nuclear RNA-seq libraries. Long nuclear RNA (>200 nt) was isolated using Zymo Clean and Concentrate columns (R1013); rRNA was removed using the Ribo-Zero rRNA removal kit (MRZH11124); and stranded libraries were prepared with the NEBNext ultra directional RNA library prep kit (E7420S). Long nuclear RNA-seq libraries were generated from two biological replicates for each tissue and were sequenced in paired-end mode. We observed that all tissue-specific libraries have noticeable background for abundant tissue-specific mRNAs (e.g., muscle myosin *unc-54*). This appears to be due at least in part by contamination by whole-animal cytoplasmic RNA released during nuclear isolation, as the RNA in the unexpected tissue is predominantly spliced.

### Data processing

Data were processed as previously described (Jänes et al. 2018) and aligned to WBcel235/ce11 genome. Further details are given in the Supplemental Methods.

To assess the reproducibility of biological replicate data sets, we used site accessibility or gene expression values to compute pairwise Euclidean distances between each data set and pairwise Pearson's correlation scores. ATAC-seq and RNA-seq biological replicates showed high concordance (Supplemental Fig. S2D).

### Classification of accessible sites

First, accessibility at each site in each sample was calculated as reads per million (RPM) values. RPMs of biological replicates were averaged to obtain a single accessibility score for each site in each tissue. Sites with accessibility <8 RPM in every tissue were not further studied.

Then, estimation of accessibility fold-changes (FCs) and adjusted *P*-values were computed between all pairs of tissues using the DESeq2 package (Love et al. 2014). A site was considered significantly differentially accessible (DA) between two tissues if there was a FC greater than three and an adjusted *P*-value < 0.01. A FC of three between consecutive tissues was used as a threshold to determine the tissue specificity of accessible sites. Classification details are provided in the Supplemental Material.

### Classification of genes

Long nuclear RNA-seq stranded fragments were assigned to *C. elegans* gene annotations (WBCel235, release 92) using the featureCounts program with “-t gene -s 2 -Q 10 -p” options. Estimation of expression FCs and adjusted *P*-values were computed between pairs of tissues using the DESeq2 package. A gene was considered significantly differentially expressed (DE) between two tissues if there was a FC greater than three and an adjusted *P*-value < 0.01.

In each sample, gene expression was calculated as transcripts per million (TPM) values. TPMs of biological duplicates were then averaged to obtain a single gene expression value for each tissue.

The rules used to classify accessible sites were also used to classify genes, with a detection threshold of 5 TPM. A small number of germline-specific genes (151) with maximal expression in L4 (Jänes et al. 2018) were classified as sperm specific and not included in this study.

### Gene Ontology (GO) analysis

GO enrichment analyses were performed using the gProfileR 0.6.7 package (Reimand et al. 2007), filtering for redundant GO terms using the hier_filtering = moderate option. To compare GO enrichment across several groups, the clusterProfiler 3.10.1 package (Yu et al. 2012) was used, filtering for redundant terms using REVIGO (Supek et al. 2011). Only GO terms with Bonferroni-adjusted *P*-values lower than 0.05 were kept.

### ATAC-seq fragment density plots

ATAC-seq fragment density plots, also known as V-plots (Henikoff et al. 2011), were generated using the VplotR package (release 0.4.0; https://github.com/js2264/VplotR). Flanking nucleosome enrichment scores were calculated from the V-plots as illustrated in Supplemental Figure S5A.

### Nucleosome occupancy tracks and +1 nucleosome mapping

Processed BAM files from paired-end ATAC-seq duplicates of each tissue or from whole organism young adults (Jänes et al. 2018) were merged. For each class of promoter (germline, neuron, muscle, hypodermis, intestine, and ubiquitous promoters), the nucleoATAC Python package (Schep et al. 2015) was used to compute the probability of nucleosome occupancy from −1 kb to +1 kb from promoter centers in each tissue (germline, neuron, muscle, hypodermis, intestine, and whole organism).

Putative +1 and −1 nucleosome positions were determined for each set of tissue-specific promoters using the corresponding tissue-specific nucleosome occupancy probability track and for ubiquitous promoters using the whole-organism nucleosome occupancy probability track (Jänes et al. 2018). We assigned the center of the putative +1 nucleosome to the local maximum of the nucleosome occupancy probability within 200 bp downstream from the forward TSS mode. Similarly, the center of the −1 nucleosome summit was assigned to the local maximum of the occupancy probability within 200 bp upstream of the reverse TSS mode. Only coding promoters with experimentally determined forward and reverse TSSs were considered.

### Motif identification and enrichment analyses

Motifs enriched in different sets of promoters (−75 bp to +105 bp from promoter centers) were identified using MEME in stranded mode and a zero-order background model (-markov_order 0). MEME mode was set to “any number of repetitions” (-mod anr), and motif widths were restricted to 6–25 bp. The five motifs found most enriched in each tissue (with an *E*-value threshold of 0.05) were retrieved. Unstranded motifs (found twice as complementary sequences, because MEME was run in stranded mode) were manually combined. PWMs for the Initiator (Inr) and the TATA motif were obtained from Jin et al. (2006). Motif mapping to promoters was performed in R v 3.5.2 (R Core Team 2019) using the Biostrings 2.50.2 package, the GenomicRanges 1.34.0 package, and the TFBSTools 1.20.0 package with a relScore threshold set to 0.8.

### Dinucleotide periodicity

To estimate dinucleotide periodicity in sets of sequences (e.g., −50 to +300 bp sequences around ubiquitous, germline, or somatic tissue–specific TSSs in Fig. 4B, or −50 to +300 bp sequences around TSSs from different organisms in Fig. 5A), the getPeriodicity() function from the periodicDNA 0.2.0 package was used with default parameters. Briefly, the distribution of distances between all possible pairs of dinucleotides in the set of sequences was computed and corrected for distance decay and smoothed by a moving average window of three, and power spectral densities were retrieved by applying a fast Fourier transform to the normalized distribution.

To generate 10-bp dinucleotide periodicity score tracks, the generatePeriodicityTrack() function from the periodicDNA package (release 0.2.0, https://github.com/js2264/periodicDNA) was used with default parameters. Briefly, a running 10-bp dinucleotide periodicity score was calculated by applying a fast Fourier transform (stats 3.5.2 package) on the distribution of distances between pairs of dinucleotides (e.g., WW……WW) found in 100-bp long sequences (2-bp increments).

### Phasing of nucleosomal sequences

To observe the 10-bp periodic occurrence of a dinucleotide in putative +1 nucleosomes, sequences (400 bp centered at the nucleosome dyads) were first clustered by *k*-means based on the dinucleotide occurrences in each sequence, and then the clusters were rephased within a −/+5-bp range using the lag value estimated by the ccf() function from the stats 3.5.2 package.

### Sets of annotations in fly, fish, mouse, and human

In worms, experimentally annotated TSSs were used (Jänes et al. 2018). In fly and zebrafish (respectively dm6 and danRer10 genome versions), TSSs were assigned to the first base of the genes using TxDb.Dmelanogaster.UCSC.dm6.ensGene 3.4.4 and TxDb.Drerio.UCSC.danRer10.refGene 3.4.4 gene models with the GenomicFeatures 1.34.7 package in R. In mouse and human, FANTOM CAGE data sets (Lizio et al. 2015) were used to retrieve the dominant TSS closest to the gene annotation. CV values were retrieved from Gerstein et al. (2014) for worm, fly, and human or computed using gene expression data sets from Pervouchine et al. (2015) for mouse and White et al. (2017) for zebrafish. Genes with the 20% lowest CVs were considered broadly expressed, and those with the 20% highest CVs were considered regulated.

### Nucleosome occupancy in fly, fish, mouse, and human

Nucleosome occupancy tracks were generated as described for worms using nucleoATAC with the following ATAC-seq data sets: SRR6171265 (Haines and Eisen 2018) in fly, SRR5398228 (Quillien et al. 2017) in zebrafish, SRR5470874 (Benchetrit et al. 2019) in mouse, and SRR891268 (Buenrostro et al. 2013) in human.

### Software availability

Processed data and all annotations are available and can be either dynamically explored or anonymously downloaded at https:// ahringerlab.com/RegAtlas/. Software for V-plotting and for analysis of periodicity of DNA motifs developed for this study are available as R packages. VplotR release 0.4.0 (Source code VplotR-0.4.0.tar.gz) is available at https:// github.com/js2264/VplotR/releases/tag/v0.4.0 and periodicDNA release 0.2.0 (Source code periodicDNA-0.2.0.tar.gz) is available at https://github.com/js2264/periodicDNA/releases/tag/v0.2.0 (both are also available as Supplemental Code).

## Data access

All raw and processed sequencing data generated in this study have been submitted to the NCBI Gene Expression Omnibus (GEO; https://www.ncbi.nlm.nih.gov/geo/) under accession number GSE141213.

## Competing interest statement

The authors declare no competing interests.

## Supplementary Material

Supplemental Material
