# Supplementary material for: Distinctive regulatory architectures of germline-active and somatic genes in C. elegans
Source: Genome Res. 2020 Dec;30(12):1752–65. doi: 10.1101/gr.265934.120 (PMC7706728; doi:10.1101/gr.265934.120)
Supplement: Supplemental Material [file supp_30_12_1752__index.html]

Distinctive regulatory architectures of germline-active and somatic genes in C. elegans — Supplemental Material 

# Distinctive regulatory architectures of germline-active and somatic genes in *C. elegans*

## Supplemental Material

- Supplemental\_Table\_S2.xlsx
- Supplemental\_Table\_S3.xlsx
- Supplemental\_Material.pdf
- VplotR-0.4.0.tar.gz.zip
- periodicDNA-0.2.0.tar.gz.zip
