# Supplementary material for: Distinctive regulatory architectures of germline-active and somatic genes in C. elegans
Source: Genome Res. 2020 Dec;30(12):1752–65. doi: 10.1101/gr.265934.120 (PMC7706728; doi:10.1101/gr.265934.120)
Supplement: Supplemental Material [file supp_gr.265934.120_Supplemental_Material.pdf]

Tissue-specific profiling reveals distinctive regulatory architectures at germline-active and somatic genes

Jacques Serizay, Yan Dong, Jürgen Jänes, Michael Chesney, Chiara Cerrato, Julie Ahringer

Supplemental methods **2**
Transgenic strains 2
Data processing 3
Annotation of new accessible sites 4
Classification of accessible sites 4
Comparison with other datasets 5
Supplemental Figures and legends **6**
Supplemental Figure S1. Reporter strains created for this study. 8
Supplemental Figure S2. Sorting strategy and dataset quality control. 10
Supplemental Figure S3. Promoter classes associated with different gene types. 12
Supplemental Figure S4. Nucleosome signatures at different types of promoters. 14
Supplemental Figure S5. Fragment density plots and flanking nucleosome enrichment scores. 16
Supplemental Figure S6. 10-bp dinucleotide periodicities at different classes of promoters. 18
Supplemental Figure S7. Location of motifs relative to ubiquitous or tissue-specific TSSs. 20
Supplemental Figure S8. RegAtlas, a web interface to explore C. elegans gene expression and chromatin accessibility datasets. 22
Supplemental Tables and Legends (Tables S2 and S3 available separately) **23**
Supplemental Table S1. Purity of sorted nuclei. 23
Supplemental Table S2. Tables of accessible sites and genes, with their tissue annotations. 23
Supplemental Table S3. Motif enrichment in sets of ubiquitous or tissue-specific promoters 24
Source code **25**
VplotR-0.4.0.zip 25
periodicDNA-0.2.0.zip 25
Supplemental References **26**

Supplemental methods

Transgenic strains

C. elegans strains were maintained using standard procedures at 25°C and fed OP50 E. coli . Targeting of the GFP to the nuclear envelope was achieved in two different ways: 1) by fusing a StrepTag (WSHPQFEK) to the N-terminal extremity of GFP (from pPD95.02, Fire lab Vector Kit) and UNC-83 (aa 1-290) to its C-terminal extremity, or 2) fusing the full-length NPP-9 coding sequence to the C-terminal extremity of GFP (Steiner et al. 2012) . The first approach was used to target GFP to the nuclear envelope in germline, muscle, hypodermis and intestine cells. The second approach was used to target GFP to the nuclear envelope in neurons. The promoter used to express the reporter in individual tissues are the *mex-5* promoter (for germline expression, chrIV:13,353,242-13,353,729), the *egl-21* promoter (for neuron expression, chrIV:10,481,768-10,481,932), the *myo-3* promoter (from muscle expression, chrV:12,234,302-12,236,686), the *dpy-7* promoter (for hypodermis expression, chrX:7,537,794-7,538,688) and the *npa-1* promoter (for intestinal expression, chrV:7,075,526-7,075,947) (coordinates are in ce11). Three-way Gateway cloning was used to clone each tissue-specific promoter (in slot 1) upstream of the reporter coding sequence (in slot 2). *tbb-2* - 3'UTR was used in slot 3 (Merritt et al. 2008). The destination vector was pCFJ150 (Frøkjaer-Jensen et al. 2008). Reporter constructs were integrated in a single copy at the ttTi5605 Mos1 site located on chr II (Frøkjaer-Jensen et al. 2008). Strain genotypes and staining patterns are in Supplemental Figure S1.

Data processing

Reads were trimmed using fastx_trimmer 0.0.14 and aligned to the reference genome WBcel235/ce11 obtained from Ensembl release 92 (ftp://ftp.ensembl.org/pub/release-92/) using bwa-backtrack 0.7.17-r1188 (Li and Durbin 2009) in single-end (ATAC-seq) or paired-end mode (ATAC-seq, long

nuclear RNA-seq). Low-quality (q < 10), mitochondrial and modENCODE-blacklisted (Consortium 2013) reads were discarded.

Normalized genome-wide accessibility tracks were computed with MACS2 (Feng et al. 2012) using parameters --format BAM --bdg --SPMR --gsize ce --nolambda --nomodel --extsize 150 --shift -75 --keep-dup all and the bedGraphToBigWig utility (Kent et al. 2010). ATAC-seq was also sequenced in paired-end mode; paired-end data were used for nucleosome occupancy and V-plots analyses (described below).

Long nuclear RNA-seq data were processed essentially as in (Chen et al. 2013). Following alignment and filtering, fragments-per-million-normalized strand-specific coverage tracks were computed by transforming the bam file into a bedGraph file using the genomeCoverageBed v2.26.0 utility (Quinlan and Hall 2010) with the parameters -bg -pc -scale 10e6/${NBFRAGS} -strand ${STRAND} (where ${NBFRAGS} is the number of mapped fragments and ${STRAND} is + or -). Gene annotations used throughout this study are WBcel235/ce11 obtained from Ensembl release 92 (ftp://ftp.ensembl.org/pub/release-92/).

Annotation of new accessible sites

In a previous study, we identified 42,245 accessible sites across development and aging and annotated them into functional classes (coding promoters, non-coding promoters, unassigned promoters, putative enhancers, inactive elements) based on nuclear RNA seq patterns (Jänes et al. 2018). The annotation pipeline of (Jänes et al. 2018) was run using the previously generated data together with the tissue-specific ATAC-seq and RNA-seq generated in this study. This resulted in the detection and annotation of 5,269 new accessible sites, bringing the total sites to 47,514. Supplemental Table S2 provides annotation of the new elements and updated annotations of the elements identified in (Jänes et al. 2018).

Classification of accessible sites

In each sample, accessibility at each site was calculated as Reads Per Million (RPM) values. RPMs of biological replicates were averaged to obtain a single accessibility score for each site in each tissue. Sites with accessibility lower than 8 RPM in every tissue were not further studied.

The tissue specificity of accessible sites was determined according to the following successive rules:

Finally, the tissue specificity of accessible sites was determined according to the following successive rules:

- *Restricted to a single tissue*: sites (i) significantly DA between the first and the second most accessible tissues and (ii) not significantly DA between the second and the third most accessible tissues.
- *Restricted to two tissues:* sites (i) significantly DA between the second and the third most accessible tissues and (ii) not significantly DA between the third and the fourth most accessible tissues.
- *Restricted to three tissues:* sites (i) significantly DA between the third and the fourth most accessible tissues and (ii) not significantly DA between the fourth and the fifth most accessible tissues.
- *Restricted to four tissues:* sites significantly DA between the fourth and the fifth most accessible tissues.
- *Ubiquitous-biased:* sites (i) significantly DA between any other pair of tissues (*e.g.* first and fourth most accessible tissue) and (ii) detected across all tissues (RPM > 8 in all replicates).
- *Ubiquitous-uniform* (also referred to as simply "uniform")*:* sites (i) not significantly DA between any pair of tissues and (ii) detected across all tissues (RPM > 8 in all replicates).

- *Unclassified:* sites with accessibility < 8 RPM in some tissues and not significantly DA could not be confidently classified.

Comparison with other datasets

Tissue-specific gene expression values from nuclear RNA-seq of sorted L4/YA nuclei were compared to those obtained by single-cell RNA-seq in L2 (Cao et al. 2017) by computing pairwise Euclidean distances between each dataset.

In Supplemental Figure 2, our gene expression classes were compared to those derived from single-cell RNA-seq in L2 (Cao et al. 2017), where genes were considered enriched in a given tissue if the expression fold-change between this tissue and the tissue with the second highest expression was higher than 5. Genes were considered detected if their expression was higher than 5 TPM in at least one tissue, and ubiquitous if (i) their expression was higher than 5 TPM across all tissues and (ii) they were not enriched in any tissue. Our gene expression classes were also compared to those obtained by tissue-specific cell sorting and RNA-seq in young adult somatic tissues (muscle, neurons, hypodermis and intestine) (Kaletsky et al. 2018).

Other visualization tools

Figures were generated in R 3.5.2 (R Core Team 2019), using either base or ggplot2 3.1.1 (Wickham 2016) plotting functions. Genome browser screenshots were obtained from IGV 2.4.8 (Robinson et al. 2011). Genome tracks in the bigWig format were imported in R using the rtracklayer 1.42.2 package (Lawrence et al. 2009).

Supplemental Figures and legends

A

Strain	Tissue	Genotype
JA1585	muscle	*unc-119(ed3); weSi52* [*Pmyo-3::* StrepTag*::GFP::unc-83(ct290aa)::tbb-2* 3'UTR; *unc-119(+)*] II
JA1616	germ line	*unc-119(ed3); weSi74* [*Pmex-5* ::StrepTag::*GFP* ::*unc-83(ct290aa*)::*tbb-2* 3'UTR; *unc-119(+)*] II
JA1815	hypodermis	*unc-119(ed3); weSi148* [*Pdpy-7* ::StrepTag::*GFP::unc-83(ct290aa)::tbb-2* 3'UTR; *unc-119(+)*] II
JA1816	neurons	*unc-119(ed3); weSi149* [*Pegl-21::npp-9::GFP::tbb-2* 3' UTR; *unc-119(+)*] II
JA1817	intestine	*unc-119(ed3); weSi150* [*Pnpa-1* ::StrepTag::*GFP::unc-83(ct290aa)::tbb-2* 3'UTR; *unc-119(+)*] II

B **C** **D** **E** **F**

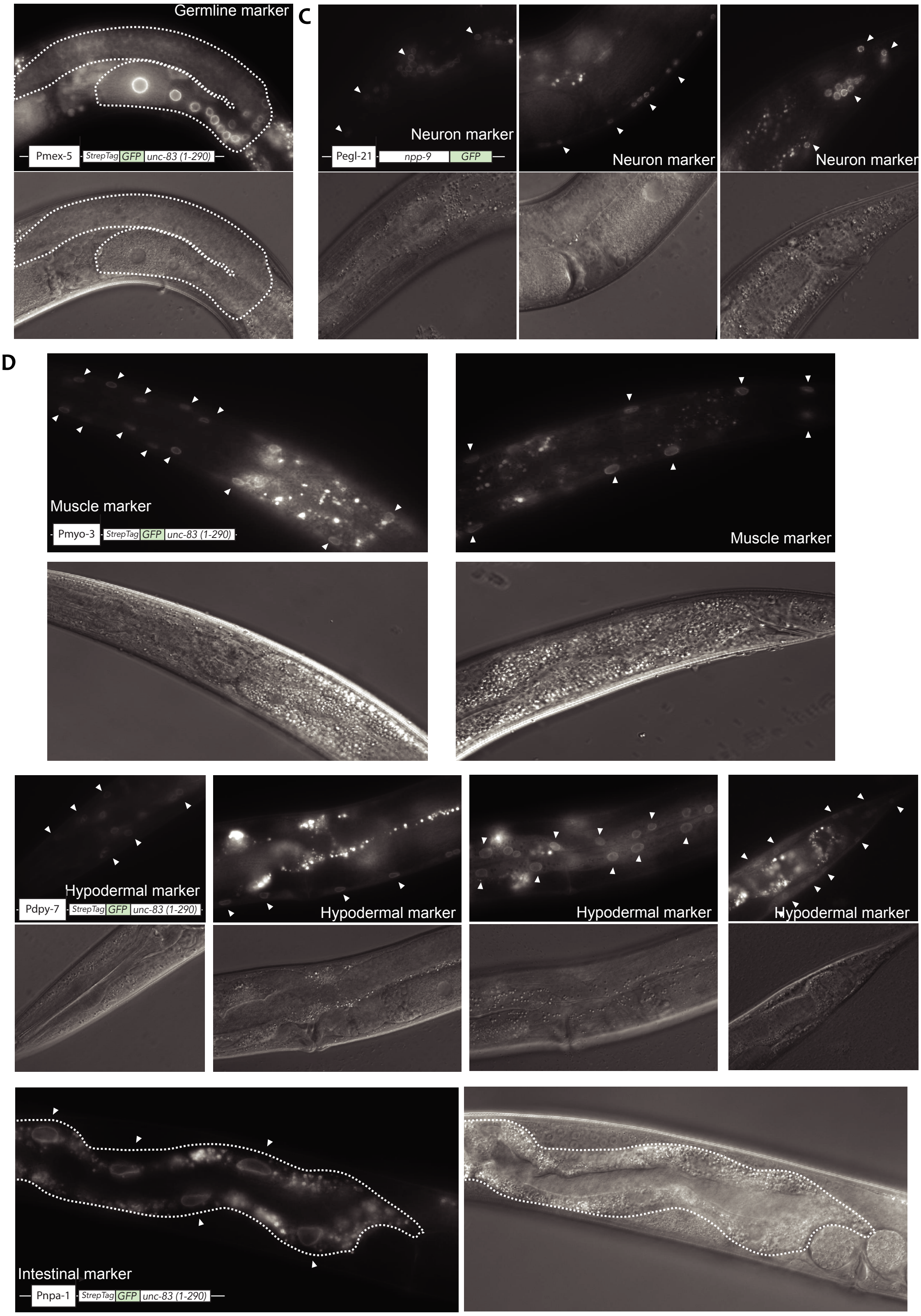

Supplemental Figure S1. Reporter strains created for this study.

(A) Strain names and genotypes. *(B-F)* Reporter strains labelling nuclear envelope of *(B)* JA1616 - germline nuclei, *(C)* JA1816 - neuronal nuclei (photos of head neurons, ventral nerve cord and tail neurons), *(D)* JA1585 -muscle nuclei (photos of anterior and posterior sides), *(E)* JA1815 - hypodermal nuclei (photos of head, ventral hypodermal ridge, seam and tail), and *(F)* JA1817 - intestinal nuclei (photos of anterior intestine). For each reporter, the construct used to drive expression of the marker is depicted. DIC images are also shown for reference (bottom).

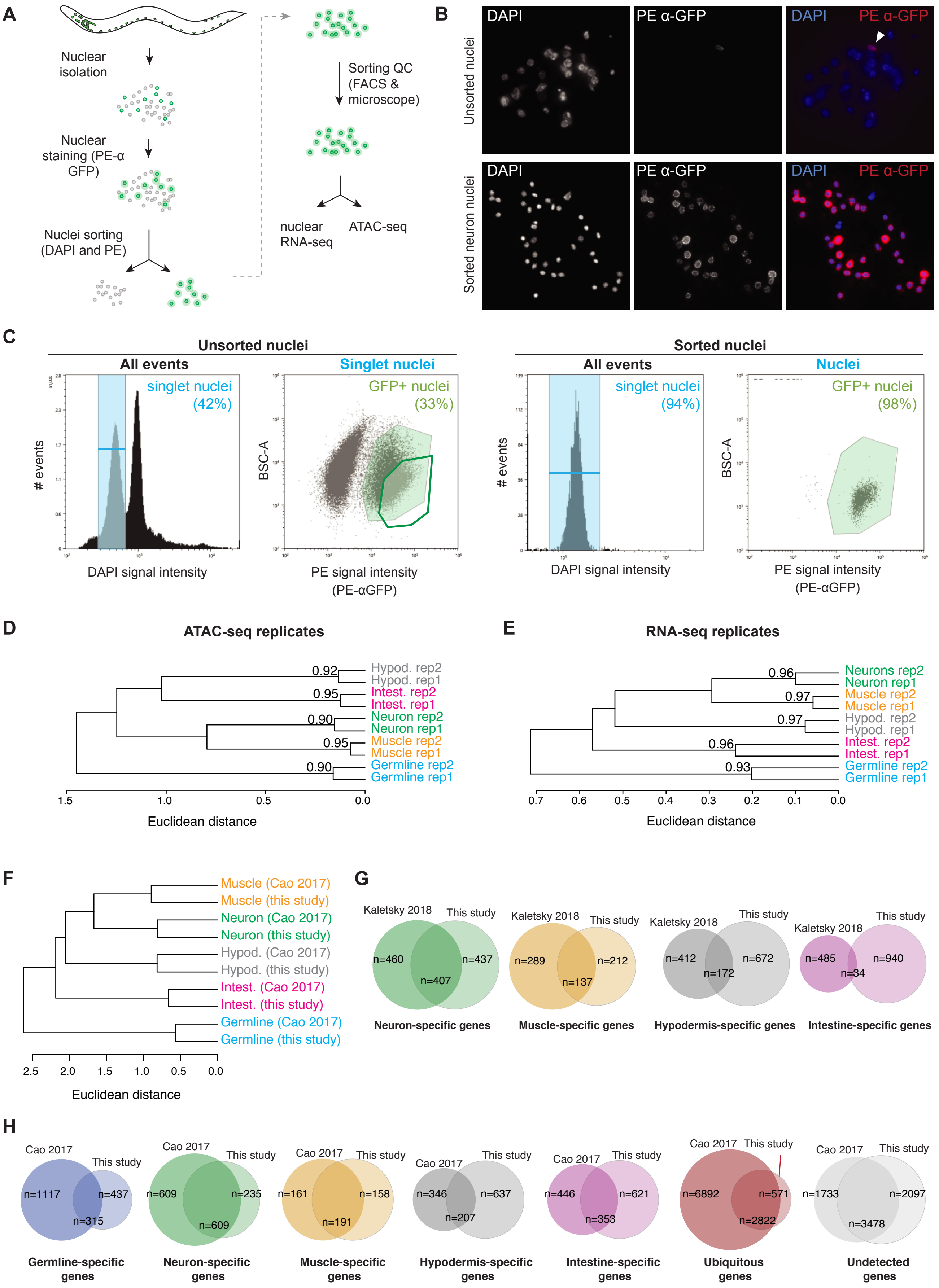
A
Nuclear isolation
Nuclear staining (PE-α GFP)
Nuclei sorting (DAPI and PE)
Sorting QC (FACS & microscope)
nuclear RNA-seq
ATAC-seq
B
Unsorted nuclei
Sorted neuron nuclei
DAPI
PE α-GFP
C
Unsorted nuclei
All events
Singlet nuclei
singlet nuclei (42%)
GFP+ nuclei (33%)
events
BSC-A
DAPI signal intensity
PE signal intensity (PE-αGFP)
Sorted nuclei
All events
Nuclei
singlet nuclei (94%)
GFP+ nuclei (98%)
D
ATAC-seq replicates
Hypod. rep2
Hypod. rep1
Intest. rep2
Intest. rep1
Neuron rep2
Neuron rep1
Muscle rep2
Muscle rep1
Germline rep2
Germline rep1
0.92
0.95
0.90
0.95
0.90
1.5 1.0 0.5 0.0
Euclidean distance
E
RNA-seq replicates
Neurons rep2
Neuron rep1
Muscle rep2
Muscle rep1
Hypod. rep2
Hypod. rep1
Intest. rep2
Intest. rep1
Germline rep2
Germline rep1
0.96
0.97
0.97
0.96
0.93
0.7 0.6 0.5 0.4 0.3 0.2 0.1 0.0
Euclidean distance
F
Muscle (Cao 2017)
Muscle (this study)
Neuron (Cao 2017)
Neuron (this study)
Hypod. (Cao 2017)
Hypod. (this study)
Intest. (Cao 2017)
Intest. (this study)
Germline (Cao 2017)
Germline (this study)
2.5 2.0 1.5 1.0 0.5 0.0
Euclidean distance
G
Kaletsky 2018
This study
n=460 n=407 n=437
Neuron-specific genes
n=289 n=137 n=212
Muscle-specific genes
n=412 n=172 n=672
Hypodermis-specific genes
n=485 n=34 n=940
Intestine-specific genes
H
Cao 2017
This study
n=1117 n=315 n=437
Germline-specific genes
n=609 n=609 n=235
Neuron-specific genes
n=161 n=191 n=158
Muscle-specific genes
n=346 n=207 n=637
Hypodermis-specific genes
n=446 n=353 n=621
Intestine-specific genes
n=6892 n=2822 n=571
Ubiquitous genes
n=1733 n=3478 n=2097
Undetected genes

Supplemental Figure S2. Sorting strategy and dataset quality control.

(A) Detailed procedure used to isolate tissue-specific nuclei. *(B)* Nuclei from neuronal reporter strain (Pegl-21::npp-9::GFP::tbb2-3'UTR) immuno-stained with a PE α-GFP antibody, before (top) and after (bottom) nuclei sorting. The arrow points to a single PE+ nucleus. *(C)* Left: gating strategy to isolate PE+ (*i.e.* GFP+) nuclei from a nuclear preparation. Single nuclei are gated (shaded blue area) and GFP+ nuclei (green shaded area) are readily separated from GFP- nuclei. Here, the gate used to sort GFP+ nuclei is the thick-lined green gate (no shading). Right: flow cytometry recording of sorted nuclei to estimate the purity of GFP+ nuclei. *(D-F)* Euclidean distances and Pearson correlation scores between ATAC-seq biological duplicates *(D)*, RNA-seq biological duplicates *(E)*, and between RNA-seq (this study) and single-cell RNA-seq from the L2 stage (Cao et al. 2017) *(F)*. *(G-H)* Intersection between gene expression annotations (this study) and those from RNA-seq in YA (Kaletsky et al. 2018) or single-cell RNA-seq in L2 (Cao et al. 2017).

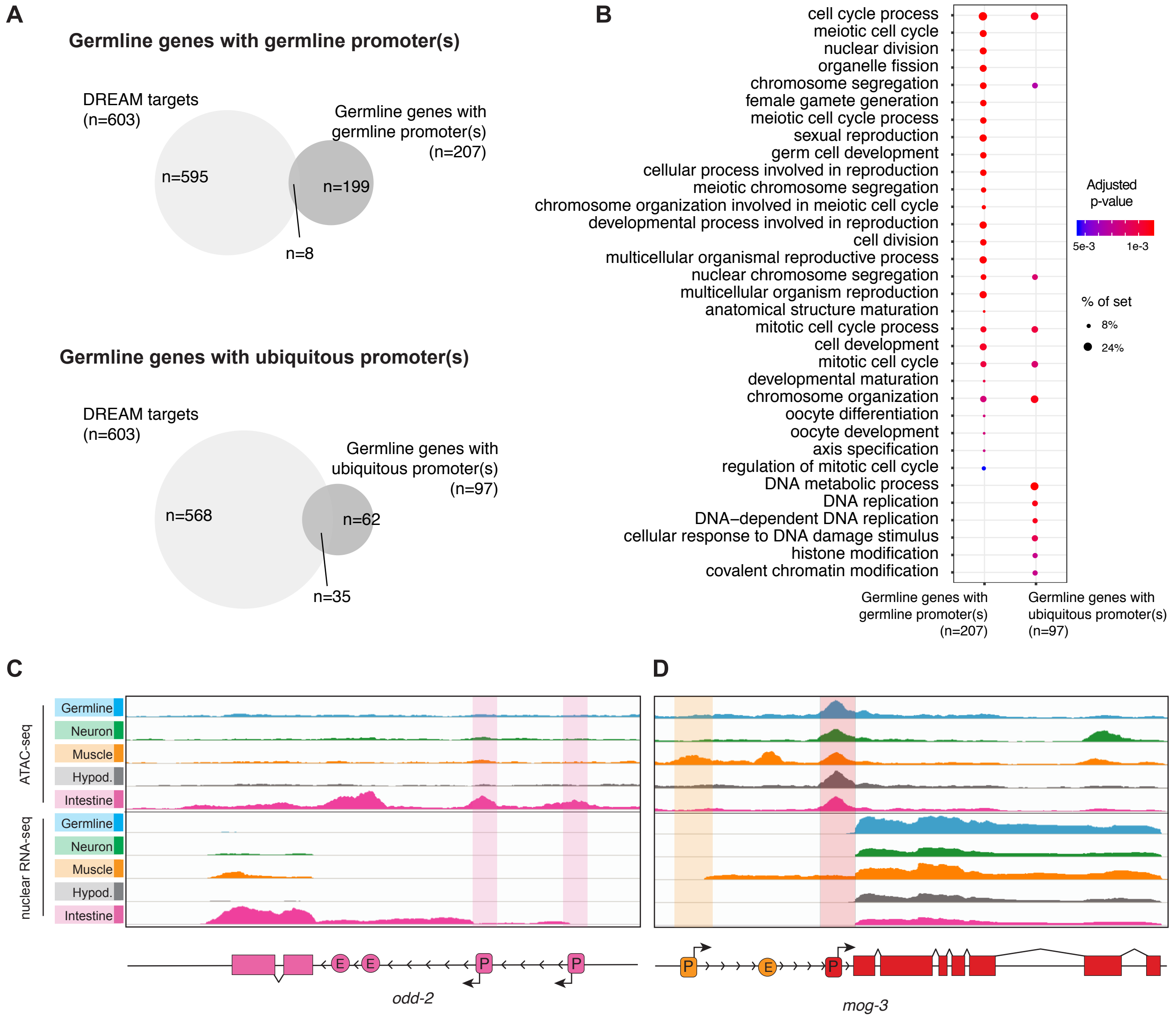
A
Germline genes with germline promoter(s)
DREAM targets (n=603)
Germline genes with germline promoter(s) (n=207)
n=595
n=199
n=8
Germline genes with ubiquitous promoter(s)
DREAM targets (n=603)
Germline genes with ubiquitous promoter(s) (n=97)
n=568
n=62
n=35
B
cell cycle process
meiotic cell cycle
nuclear division
organelle fission
chromosome segregation
female gamete generation
meiotic cell cycle process
sexual reproduction
germ cell development
cellular process involved in reproduction
meiotic chromosome segregation
chromosome organization involved in meiotic cell cycle
developmental process involved in reproduction
cell division
multicellular organismal reproductive process
nuclear chromosome segregation
multicellular organism reproduction
anatomical structure maturation
mitotic cell cycle process
cell development
mitotic cell cycle
developmental maturation
chromosome organization
oocyte differentiation
oocyte development
axis specification
regulation of mitotic cell cycle
DNA metabolic process
DNA replication
DNA−dependent DNA replication
cellular response to DNA damage stimulus
histone modification
covalent chromatin modification
Germline genes with germline promoter(s) (n=207)
Germline genes with ubiquitous promoter(s) (n=97)
Adjusted p-value
5e-3
1e-3
% of set
8%
24%
C
ATAC-seq
nuclear RNA-seq
Germline
Neuron
Muscle
Hypod.
Intestine
E
P
odd-2
D
mog-3

Supplemental Figure S3. Promoter classes associated with different gene types.

(A) Intersection of DREAM targets defined in (Latorre et al. 2015) with germline genes with only germline-specific promoter(s) (top) or only ubiquitous promoter(s) (bottom). *(B)* GO terms enriched in germline genes with only germline-specific or only ubiquitous promoter(s). *(C)* Example of a tissue-specific gene with multiple tissue-specific promoters (here *odd-2*, an intestine gene with two intestine-specific promoters). *(D)* Example of a ubiquitous gene with a ubiquitous promoter and a tissue-specific promoter (here *mog-3*, with one ubiquitous and one muscle-specific promoter).

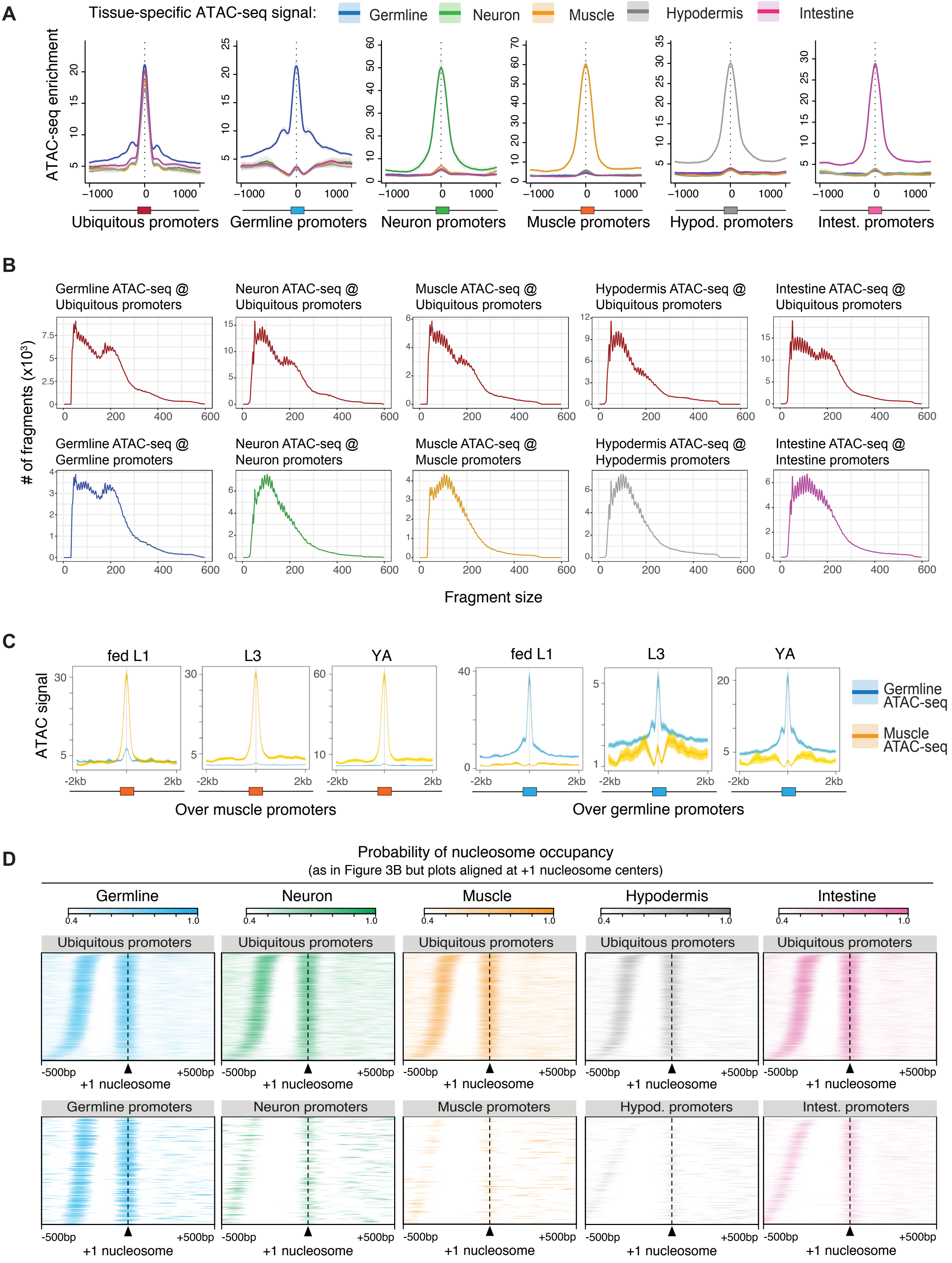
A
Tissue-specific ATAC-seq signal:
Germline
Neuron
Muscle
Hypodermis
Intestine
ATAC-seq enrichment
Ubiquitous promoters
Germline promoters
Neuron promoters
Muscle promoters
Hypod. promoters
Intest. promoters
B
Germline ATAC-seq @ Ubiquitous promoters
Neuron ATAC-seq @ Ubiquitous promoters
Muscle ATAC-seq @ Ubiquitous promoters
Hypodermis ATAC-seq @ Ubiquitous promoters
Intestine ATAC-seq @ Ubiquitous promoters
Germline ATAC-seq @ Germline promoters
Neuron ATAC-seq @ Neuron promoters
Muscle ATAC-seq @ Muscle promoters
Hypodermis ATAC-seq @ Hypodermis promoters
Intestine ATAC-seq @ Intestine promoters
# of fragments (x10³)
Fragment size
C
fed L1
L3
YA
ATAC signal
Germline ATAC-seq
Muscle ATAC-seq
Over muscle promoters
Over germline promoters
D
Probability of nucleosome occupancy
(as in Figure 3B but plots aligned at +1 nucleosome centers)
Germline
Neuron
Muscle
Hypodermis
Intestine
Ubiquitous promoters
Germline promoters
Neuron promoters
Muscle promoters
Hypod. promoters
Intest. promoters
-500bp
+500bp
+1 nucleosome

Supplemental Figure S4. Nucleosome signatures at different types of promoters.

(A) Metaplots of tissue-specific ATAC-seq tracks over different classes of promoters. *(B)* Size distribution of ATAC-seq fragments from different tissue-specific datasets, mapping over ubiquitous or tissue-specific promoters. *(C)* Metaplots of germline and muscle-specific ATAC-seq tracks obtained at multiple developmental stages (L1, L3 and L4/YA) over germline or muscle-specific promoters. *(D)* Same figure as in Fig. 3B, but with nucleosome occupancy signals centered at +1 nucleosome summits rather than at TSSs. Rows are ordered by NDR widths.

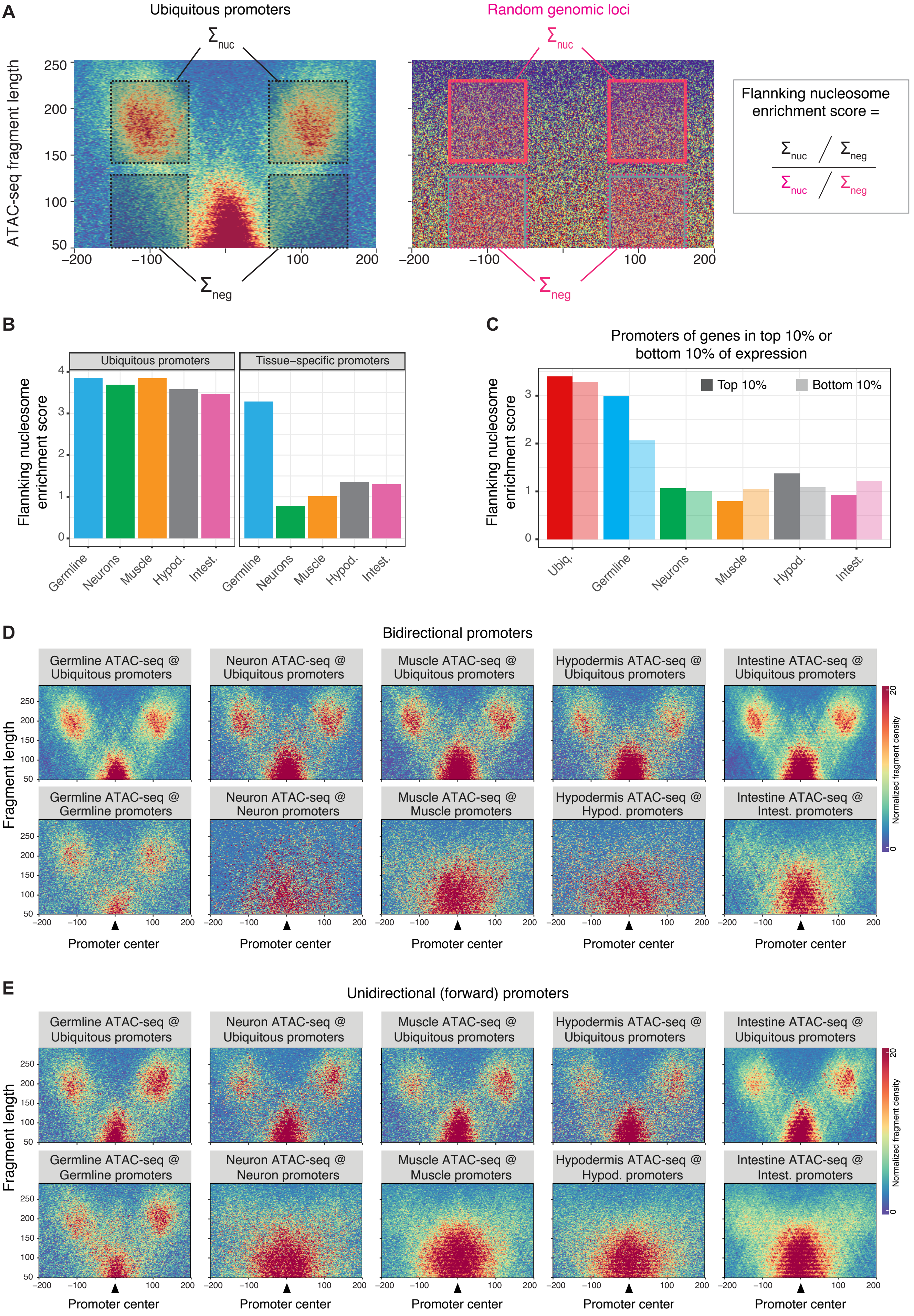
A
Ubiquitous promoters
Random genomic loci
ATAC-seq fragment length
Σnuc
Σneg
Flannking nucleosome enrichment score =
Σnuc / Σneg
Σnuc / Σneg
B
Ubiquitous promoters
Tissue–specific promoters
Flannking nucleosome enrichment score
Germline
Neurons
Muscle
Hypod.
Intest.
C
Promoters of genes in top 10% or bottom 10% of expression
Top 10%
Bottom 10%
Ubiq.
D
Bidirectional promoters
Germline ATAC-seq @ Ubiquitous promoters
Neuron ATAC-seq @ Ubiquitous promoters
Muscle ATAC-seq @ Ubiquitous promoters
Hypodermis ATAC-seq @ Ubiquitous promoters
Intestine ATAC-seq @ Ubiquitous promoters
Germline ATAC-seq @ Germline promoters
Neuron ATAC-seq @ Neuron promoters
Muscle ATAC-seq @ Muscle promoters
Hypodermis ATAC-seq @ Hypod. promoters
Intestine ATAC-seq @ Intest. promoters
Fragment length
Promoter center
Normalized fragment density
E
Unidirectional (forward) promoters
Germline ATAC-seq @ Ubiquitous promoters
Neuron ATAC-seq @ Ubiquitous promoters
Muscle ATAC-seq @ Ubiquitous promoters
Hypodermis ATAC-seq @ Ubiquitous promoters
Intestine ATAC-seq @ Ubiquitous promoters
Germline ATAC-seq @ Germline promoters
Neuron ATAC-seq @ Neuron promoters
Muscle ATAC-seq @ Muscle promoters
Hypodermis ATAC-seq @ Hypod. promoters
Intestine ATAC-seq @ Intest. promoters
Fragment length
Promoter center
Normalized fragment density

Supplemental Figure S5. Fragment density plots and flanking nucleosome enrichment scores.

(A) Method to compute flanking nucleosome enrichment scores from ATAC-seq fragment density plots. *(B)* Flanking nucleosome enrichment scores at ubiquitous or tissue-specific promoters in different tissues,. *(C)* Flanking nucleosome enrichment scores at promoters associated with either the 10% most highly expressed tissue-specific genes (dark bars) or the 10% most lowly expressed tissue-specific genes (light bars). Note that promoters of both highly and lowly expressed ubiquitous and germline-specific genes have an enriched +1 nucleosome whereas promoters of soma-restricted genes do not, irrespective of their expression level. *(D-E)* V-plots over *(D)* bidirectional promoters or *(E)* unidirectional (forward) promoters.

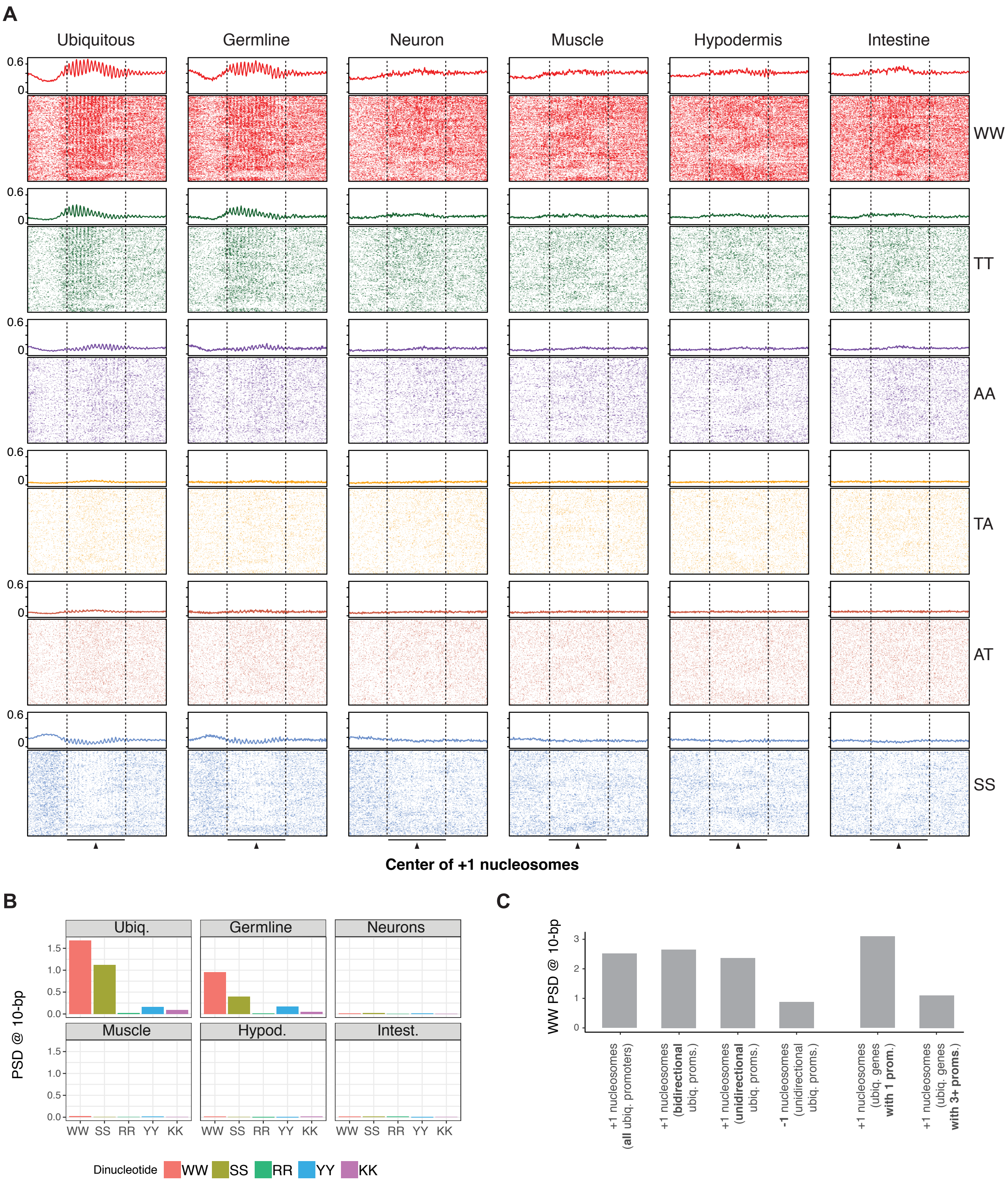

A
Ubiquitous
Germline
Neuron
Muscle
Hypodermis
Intestine
0.6
0
WW
TT
AA
TA
AT
SS
Center of +1 nucleosomes
B
Ubiq.
Germline
Neurons
Muscle
Hypod.
Intest.
PSD @ 10-bp
1.5
1.0
0.5
0.0
WW SS RR YY KK
Dinucleotide WW SS RR YY KK
C
WW PSD @ 10-bp
3
2
1
0
+1 nucleosomes (all ubiq. promoters)
+1 nucleosomes (bidirectional ubiq. proms.)
+1 nucleosomes (unidirectional ubiq. proms.)
-1 nucleosomes (unidirectional ubiq. proms.)
+1 nucleosomes (ubiq. genes with 1 prom.)
+1 nucleosomes (ubiq. genes with 3+ proms.)

Supplemental Figure S6. 10-bp dinucleotide periodicities at different classes of promoters. *(A)* WW, TT, AA, TA, AT and SS dinucleotide occurrences observed at +1 nucleosomes of ubiquitous or tissue-specific promoters (400 bp window centered at nucleosome dyads). Rows were shifted up to 5 bp to highlight the phased 10-bp periodic patterns. Summed dinucleotide occurrences are represented on top of each heatmap by a line plot. *(B)* Power spectral density (PSD) values at a 10-bp period for different dinucleotides in +1 nucleosome sequences of ubiquitous and tissue-specific promoters. *(C)* WW PSD values at a 10-bp period at +1 nucleosomes of different sets of ubiquitous promoters and at -1 nucleosomes of unidirectional ubiquitous promoters.

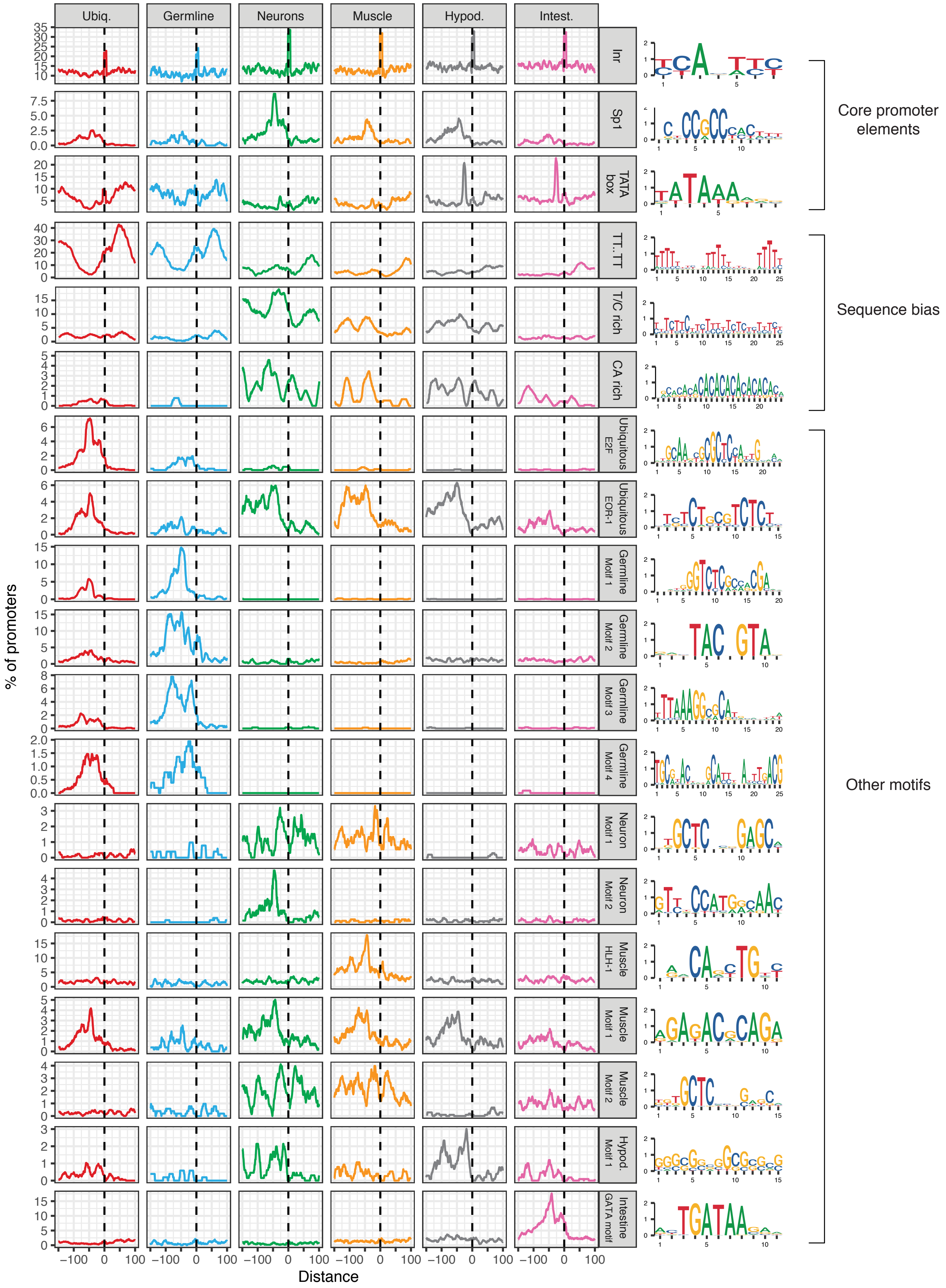

Ubiq.
Germline
Neurons
Muscle
Hypod.
Intest.
Inr
Sp1
TATA box
TT..TT
T/C rich
CA rich
Ubiquitous E2F
Ubiquitous EOR-1
Germline Motif 1
Germline Motif 2
Germline Motif 3
Germline Motif 4
Neuron Motif 1
Neuron Motif 2
Muscle HLH-1
Muscle Motif 1
Muscle Motif 2
Hypod. Motif 1
Intestine GATA motif
% of promoters
Distance
Core promoter elements
Sequence bias
Other motifs

Supplemental Figure S7. Location of motifs relative to ubiquitous or tissue-specific TSSs. Motif PWMs are displayed on the right. Only promoters with experimentally defined TSSs were considered.

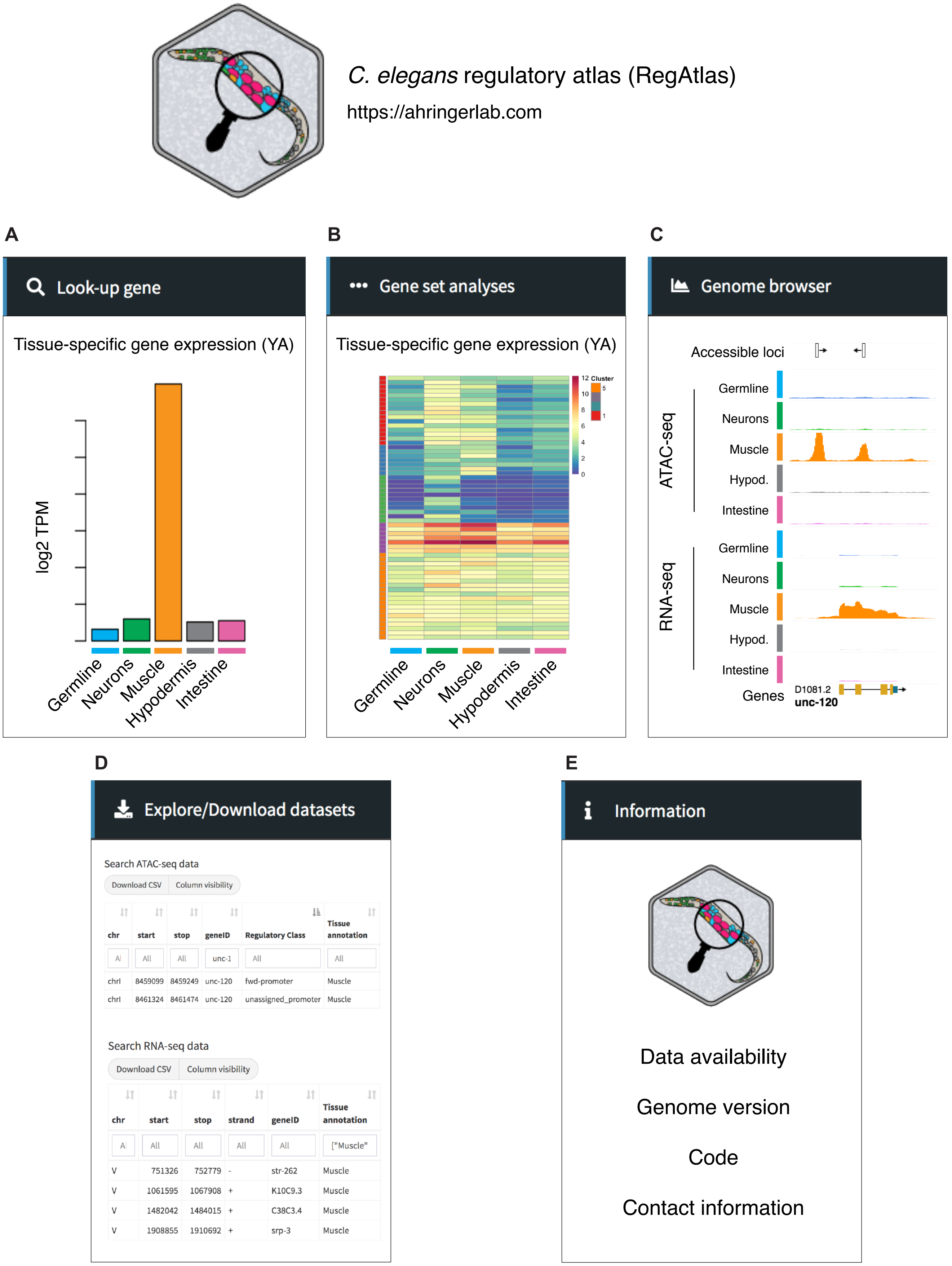

C. elegans regulatory atlas (RegAtlas)
https://ahringerlab.com
A
Look-up gene
Tissue-specific gene expression (YA)
log2 TPM
Germline
Neurons
Muscle
Hypodermis
Intestine
B
Gene set analyses
Tissue-specific gene expression (YA)
Cluster
Germline
Neurons
Muscle
Hypodermis
Intestine
C
Genome browser
Accessible loci
ATAC-seq
RNA-seq
Germline
Neurons
Muscle
Hypod.
Intestine
Genes
D1081.2
unc-120
D
Explore/Download datasets
Search ATAC-seq data
Download CSV
Column visibility
chr start stop geneID Regulatory Class Tissue annotation
chrI 8459099 8459249 unc-120 fwd-promoter Muscle
chrI 8461324 8461474 unc-120 unassigned_promoter Muscle
Search RNA-seq data
Download CSV
Column visibility
chr start stop strand geneID Tissue annotation
V 751326 752779 - str-262 Muscle
V 1061595 1067908 + K10C9.3 Muscle
V 1482042 1484015 + C38C3.4 Muscle
V 1908855 1910692 + srp-3 Muscle
E
Information
Data availability
Genome version
Code
Contact information

Supplemental Figure S8. RegAtlas, a web interface to explore *C. elegans* gene expression and chromatin accessibility datasets.

Interface of RegAtlas, a web application developed to explore developmental and tissue-specific genomic datasets. RegAtlas is hosted at https://ahringerlab.com. Its use is entirely anonymous and performed queries are not saved. *(A)* Tab to query information on a single gene. *(B)* Tab to intersect a user-provided list of genes with tissue-specific and ubiquitous sets of genes defined in this study, visualize their expression across development or in adult tissues and perform GO enrichment analysis. *(C)* Tab to dynamically browse different types of genomic tracks (*e.g.* developmental or tissue-specific ATAC-seq and RNA-seq tracks) using an integrated JBrowse genome browser (Buels et al. 2016). *(D)* Tab to explore and download all processed datasets in tables. *(E)* An information tab is also available to get more details about the web portal.

Supplemental Tables and legends (files available separately)

Supplemental Table S1. Purity of sorted nuclei.

For each sample, the purity of the sorted nuclei was estimated by re-running the sample through a flow cytometer. The % values represent the proportion of PE+ nuclei in the sorted nuclei. Purity of samples indicated by a star was estimated by counting ~50 nuclei under the microscope.

	ATAC-seq rep1	ATAC-seq rep2	RNA-seq rep1	RNA-seq rep2
Germline	96%	97%	97%	98%
Neurons	97%	98%	95%	98%
Muscle	100%	*98%**	*97%**	*98%**
Hypodermis	97%	96%	99%	*95%**
Intestine	97%	98%	99%	98%

Supplemental Table S2. Tables of accessible sites and genes, with their tissue annotations.

Mapping and annotation of accessible sites was performed as described in (Jänes et al. 2018). ATAC_metrics tab: (columns 1-3) ce11 chromosome, start and stop coordinates of accessible site. (column 4) accessible site type, obtained by combining strand-specific annotations. (columns 5-6) forward and reverse annotation of accessible sites. (columns 7-9) associated gene ID, locus ID and gene biotype for sites annotated as forward coding_promoter, pseudogene_promoter or non-coding_RNA in column 5. (columns 10-12) associated gene ID, locus ID and gene biotype for sites annotated as reverse coding_promoter, pseudogene_promoter or non-coding_RNA in column 6. (columns 13-14) Associated gene ID and locus ID for sites annotated as unassigned_promoter, putative_enhancer or other_element (in column 4) which overlap a gene body or outron. If a site overlaps multiple genes, all overlaps are reported, separated by commas. (columns 15-16) ce11

forward and reverse transcription initiation mode. (column 17) Newly annotated accessible sites. (columns 18-22) Average accessibility (RPM) in each tissue in L4/YAs. (columns 23-27) Tissues ranked by their expression. (columns 28-31) Ratios of gene expression between consecutive tissues. (column 32) Tissue annotation. RNA_metrics tab: (columns 1-4) ce11 chromosome, start and stop coordinates and strand of genes. (columns 5-6) Gene WormBase ID and locus ID (Ensembl release 92). (columns 7-8) Number of associated promoters and enhancers. (columns 9-13) Average expression (TPM) in each tissue in young adults. (columns 14-18) Tissues ranked by their expression. (columns 19-22) Ratios of gene expression between consecutive tissues. (column 23) Tissue annotation.

Supplemental Table S3. Motif enrichment in sets of ubiquitous or tissue-specific promoters

(column 1) Motif name from Supplemental Fig S7. (column 2) Promoter class. (column 3) Range around TSS in which the motif was counted. (column 4) Number of promoters harboring the motif in the range indicated in column 3. (column 5) Number of promoters without the motif. (column 6) Enrichment score of motif in the promoter class compared to other promoter classes (Odds ratio from Fisher's exact test). (column 7) multiple testing corrected p-values (FDR from one-sided Fisher's exact tests).

Source code

VplotR-0.4.0.zip

VplotR release 0.4.0 has been used to perform analyses in this publication. It is available at

https://github.com/js2264/VplotR/releases/tag/v0.4.0.

periodicDNA-0.2.0.zip

periodicDNA release 0.2.0 has been used to perform analyses in this publication. It is available at

https://github.com/js2264/periodicDNA/releases/tag/v0.2.0.

Supplemental References

Buels R, Yao E, Diesh CM, Hayes RD, Munoz-Torres M, Helt G, Goodstein DM, Elsik CG, Lewis SE, Stein L, et al. 2016. JBrowse: a dynamic web platform for genome visualization and analysis. *Genome Biol* **17**: 66.

Cao J, Packer JS, Ramani V, Cusanovich DA, Huynh C, Daza R, Qiu X, Lee C, Furlan SN, Steemers FJ, et al. 2017. Comprehensive single-cell transcriptional profiling of a multicellular organism. *Science* **357**: 661–667.

Chen RA-J, Down TA, Stempor P, Chen QB, Egelhofer TA, Hillier LW, Jeffers TE, Ahringer J. 2013. The landscape of RNA polymerase II transcription initiation in C. elegans reveals promoter and enhancer architectures. *Genome Res* **23**: 1339–1347.

Consortium EP. 2013. An integrated encyclopedia of DNA elements in the human genome. *Nature* **489**: 57–74.

Feng J, Liu T, Qin B, Zhang Y, Liu XS. 2012. Identifying ChIP-seq enrichment using MACS. *Nat Protoc* **7**: 1728–1740.

Frøkjaer-Jensen C, Davis MW, Hopkins CE, Newman BJ, Thummel JM, Olesen S-P, Grunnet M, Jorgensen EM. 2008. Single-copy insertion of transgenes in Caenorhabditis elegans. *Nat Genet* **40**: 1375–1383.

Jänes J, Dong Y, Schoof M, Serizay J, Appert A, Cerrato C, Woodbury C, Chen R, Gemma C, Huang N, et al. 2018. Chromatin accessibility dynamics across C. elegans development and ageing. *Elife* **7**. http://dx.doi.org/10.7554/eLife.37344.

Kaletsky R, Yao V, Williams A, Runnels AM, Tadych A, Zhou S, Troyanskaya OG, Murphy CT. 2018. Transcriptome analysis of adult Caenorhabditis elegans cells reveals tissue-specific gene and isoform expression. *PLoS Genet* **14**: e1007559.

Kent WJ, Zweig AS, Barber G, Hinrichs AS, Karolchik D. 2010. BigWig and BigBed: enabling browsing of large distributed datasets. *Bioinformatics* **26**: 2204–2207.

Latorre I, Chesney MA, Garrigues JM, Stempor P, Appert A, Francesconi M, Strome S, Ahringer J. 2015. The DREAM complex promotes gene body H2A.Z for target repression. *Genes and Development* **29**: 495–500.

Lawrence M, Gentleman R, Carey V. 2009. rtracklayer: an R package for interfacing with genome browsers. *Bioinformatics* **25**: 1841–1842. http://bioinformatics.oxfordjournals.org/content/25/14/1841.abstract.

Li H, Durbin R. 2009. Fast and accurate short read alignment with Burrows–Wheeler transform. *Bioinformatics* **25**: 1754–1760.

Merritt C, Rasoloson D, Ko D, Seydoux G. 2008. 3′ UTRs Are the Primary Regulators of Gene Expression in the C. elegans Germline. *Curr Biol* **18**: 1476–1482.

Quinlan AR, Hall IM. 2010. BEDTools: a flexible suite of utilities for comparing genomic features.

Bioinformatics **26**: 841–842.

R Core Team. 2019. R: A Language and Environment for Statistical Computing. https://www.R-project.org/.

Robinson JT, Thorvaldsdóttir H, Winckler W, Guttman M, Lander ES, Getz G, Mesirov JP. 2011. Integrative genomics viewer. *Nat Biotechnol* **29**: 24–26.

Steiner FA, Talbert PB, Kasinathan S, Deal RB, Henikoff S. 2012. Cell-type-specific nuclei purification from whole animals for genome-wide expression and chromatin profiling. *Genome Res* **22**: 766–777.

Wickham H. 2016. ggplot2: Elegant Graphics for Data Analysis. https://ggplot2.tidyverse.org.
