# Supplementary material for: Distinctive regulatory architectures of germline-active and somatic genes in C. elegans
Source: Genome Res. 2020 Dec;30(12):1752–65. doi: 10.1101/gr.265934.120 (PMC7706728; doi:10.1101/gr.265934.120)
Supplement: Supplemental Material [file supp_gr.265934.120_VplotR-0.4.0.tar.gz.zip › VplotR-0.4.0.tar.gz!/VplotR-0.4.0/docs/articles/VplotR.html]

VplotR • VplotR
 


  


 


Toggle navigation


VplotR
0.4.0

- Get started
- Reference
- Articles
  - In-depth use

# VplotR

#### Jacques Serizay

#### 2020-02-09

`VplotR.Rmd`

VplotR

## Introduction

This R package makes the process of generating fragment density plots (also known as “V-plots”) straightforward.  
V-plots have been introduced for the first time by the Henikoff lab in 2011. Recently, V-plots have proven to be very instructive to understand the molecular organization of the chromatin. For instance, the nucleoATAC package relies on cross-correlation of ATAC-seq fragment density plots to accurately map nucleosome occupancy along the genome.  
VplotR aim is to streamline the process of generating V-plots. It contains wrapping functions to import paired-end sequencing bam files and generate V-plots around genomic loci of interest.  
VplotR is designed around ggplot2 and makes full use of its potential. As such, it is easy to generate V-plots in batches and combine them with other plots to make publication-ready figures.

## Installation

VplotR can be installed from Github as follows:

```
install.packages("devtools")
devtools::install_github("js2264/VplotR")
library(VplotR)
```

## Quick V-plot

Firstly BAM files are read using the `importPEBamFiles()` function and loci of interest from a BED file, for instance.

```
granges <- rtracklayer::import('loci_of_interest.bed')
fragments <- importPEBamFiles(
    'ATAC-seq-mapped-fragments.bam', 
    where = GenomicRanges::resize(granges, width = 2000, fix = 'center'), 
    shift_ATAC_fragments = TRUE
)
```

*Note: to allow for a background normalization, the `where` argument should be omitted.*

Then V-plot of ATAC-seq fragments (`fragments`) over the loci of interest (`granges`) is generated using the `plotVmat()` function:

```
plotVmat(fragments, granges)
```

The generation of multiple V-plots can be parallelized as follow:

```
list_params <- list(
    "sample_1" = list("bam" = fragments_1, "granges" = granges_1), 
    "sample_2" = list("bam" = bam_2, "granges" = granges_2), 
    ..., 
    "sample_N" = list("bam" = bam_N, "granges" = granges_N)
)
plotVmat(
    list_params, 
    cores = length(list_params)
) + ggplot2::facet_wrap(~Cond.)
```

Finally, the `nucleosomeEnrichment()` function is useful to statistically quantify and compare nucleosome enrichment (e.g. flanking nucleosome at promoters). To do so:

```
nuc_enrich <- nucleosomeEnrichment(
    bam_granges = fragments, 
    granges = granges
)
```

For more details and additional functions, read the Introduction vignette.

### Building package

This package was built by executing the following commands in R:

```
devtools::document()
devtools::build()
pkgdown::build_site()
```

### Session Info

```
#> R version 3.5.2 (2018-12-20)
#> Platform: x86_64-pc-linux-gnu (64-bit)
#> Running under: Ubuntu 18.04.2 LTS
#> 
#> Matrix products: default
#> BLAS: /usr/lib/x86_64-linux-gnu/blas/libblas.so.3.7.1
#> LAPACK: /usr/lib/x86_64-linux-gnu/lapack/liblapack.so.3.7.1
#> 
#> locale:
#>  [1] LC_CTYPE=en_GB.UTF-8       LC_NUMERIC=C              
#>  [3] LC_TIME=en_GB.UTF-8        LC_COLLATE=en_GB.UTF-8    
#>  [5] LC_MONETARY=en_GB.UTF-8    LC_MESSAGES=en_GB.UTF-8   
#>  [7] LC_PAPER=en_GB.UTF-8       LC_NAME=C                 
#>  [9] LC_ADDRESS=C               LC_TELEPHONE=C            
#> [11] LC_MEASUREMENT=en_GB.UTF-8 LC_IDENTIFICATION=C       
#> 
#> attached base packages:
#> [1] stats     graphics  grDevices utils     datasets  methods   base     
#> 
#> loaded via a namespace (and not attached):
#>  [1] Rcpp_1.0.1       rprojroot_1.3-2  crayon_1.3.4     digest_0.6.18   
#>  [5] assertthat_0.2.1 MASS_7.3-51.1    R6_2.4.0         backports_1.1.3 
#>  [9] magrittr_1.5     evaluate_0.13    stringi_1.3.1    rlang_0.4.2     
#> [13] fs_1.3.1         rmarkdown_1.12.6 pkgdown_1.4.1    desc_1.2.0      
#> [17] tools_3.5.2      stringr_1.4.0    yaml_2.2.0       xfun_0.5        
#> [21] compiler_3.5.2   memoise_1.1.0    htmltools_0.3.6  knitr_1.22
```

## Contents

- Introduction
- Installation
- Quick V-plot
  - Building package
  - Session Info

Developed by Jacques Serizay.

Site built with pkgdown 1.4.1.
