# Supplementary material for: Distinctive regulatory architectures of germline-active and somatic genes in C. elegans
Source: Genome Res. 2020 Dec;30(12):1752–65. doi: 10.1101/gr.265934.120 (PMC7706728; doi:10.1101/gr.265934.120)
Supplement: Supplemental Material [file supp_gr.265934.120_VplotR-0.4.0.tar.gz.zip › VplotR-0.4.0.tar.gz!/VplotR-0.4.0/docs/articles/advanced.html]

VplotR advanced functions • VplotR
 


  


 


Toggle navigation


VplotR
0.4.0

- Get started
- Reference
- Articles
  - In-depth use

# VplotR advanced functions

#### Jacques Serizay

#### 2020-02-09

`advanced.Rmd`

## 0. Set up the session

```
require(tidyverse)
require(VplotR)
require(ggplot2)
```

## 1. Import reads bam files and genomic loci files

We can import .bam files of interest from a given folder using the `importBamFiles()` function.

```
# Import bam file over annotated promoters, from a local folder. 
# WARNING: This takes quite a long time for libraries that are deeply sequenced
bam_files <- list.files('~/path/to/_bam-files/')
bam_list <- importPEBamFiles(
    bam_files, 
    shift_ATAC_fragments = TRUE
)
names(bam_list) <- bam_files
```

For this vignette, relevant data is also hosted on the Ahringer server:

```
# Import ATAC-seq fragments 
bam_list <- readRDS(url('http://ahringerlab.com/VplotR/ATAC_PE_fragments.rds'))
lengths(bam_list)
```

We also need some relevant genomic locations. Let’s get different sets of *C. elegans* promoters.

```
# Import annotated ce11 promoters 
proms_list <- readRDS(url('http://ahringerlab.com/VplotR/promoters_Granges.rds'))
lengths(proms_list)
```

## 2. Check distribution of fragment sizes over a specific set of segments

Let’s have a look at the distribution of fragment sizes in each ATAC-seq library, over the different classes of promoters we have. For this, we can use the `getFragmentsDistribution()` function.

```
# For each bam file, plot its distribution of fragment sizes over the tissue-specific REs or ubiquitous REs
sizes <- parallel::mclapply(names(bam_list), function(TISSUE) {
    getFragmentsDistribution(bam_list[[TISSUE]], proms_list, roll = 5)
}, mc.cores = length(bam_list)) %>%
    setNames(names(bam_list)) %>%
    namedListToLongFormat() %>% 
    setColNames(c('REs', 'Fragment_size', 'Number', 'Promoters', 'Sample')) %>%
    dplyr::mutate(Promoters = factor(Promoters, levels = names(proms_list))) %>% 
    dplyr::mutate(max_number = unlist(lapply(levels(Sample), function(TISSUE) {
        rep(max(Number[Sample == TISSUE]), sum(Sample == TISSUE))
    }))) %>% 
    dplyr::filter(Sample != 'mixed') %>% 
    dplyr::filter(Promoters == 'Ubiq._proms' | (gsub('_proms', '', .$Promoters) == .$Sample))
# Plot results
ggplot(sizes, aes(x = Fragment_size, y = Number, color = Promoters)) +
    geom_blank(aes(y = max_number)) + 
    geom_line() +
    theme_bw() +
    labs(
        title = 'Distribution of fragment sizes', 
        x = 'Fragment size',
        y = '# of fragments',
        color = 'Promoters classes'
    ) + 
    facet_wrap(Sample~Promoters, scales = "free") + 
    scale_color_manual(
        values = c('#991919', '#1232D9', '#3B9B46', '#D99B12', '#9e9e9e', '#D912D4')
    )
```

We can already notice that there is a lack of second/third/etc. nucleosome “bump” in somatic-specific ATAC-seq data over somatic REs. However, a more in-depth view of nucleosome binding would be desirable, to better understand the molecular context of promoter organization.

## 3. Vplots

### 3.1 Quick Vplot

Particularly, *where* fragments are mapping is an information as important as *how long* they are. The fragment length distribution plots generated hereabove do not show the former parameter (where each fragment is mapping). For this reason, we created a function to generate V-plots, to show distribution of ATAC fragments of variable sizes over a set of regions of interest. Such plot shows:

- On the x axis: the position of the ATAC fragments (relative to the center of the loci of interest);
- On the y axis: the size of the ATAC fragments

```
Vmat <- bam_list[['Neurons']] %>% 
    computeVmat(proms_list[['Neurons_proms']]) %>%
    normalizeVmat(normFun = 'pctsum', roll = 3) %>% 
    plotVmat(
        main = 'Neurons-spe. ATAC fragments over Neurons-spe. proms'
    )
ggsave('examples/Vmat_Neurons-over-neurons-REs.pdf')
# Or do all at once in one function:
neurons_atac_neurons_proms <- plotVmat(
    bam_list[['Neurons']], 
    proms_list[['Neurons_proms']], 
    estimate_background = FALSE, 
    main = 'Neurons-spe. ATAC fragments over Neurons-spe. proms', 
)
gl_atac_gl_proms <- plotVmat(
    bam_list[['Germline']], 
    proms_list[['Germline_proms']], 
    estimate_background = FALSE, 
    main = 'Germline-spe. ATAC fragments over Germline-spe. proms', 
)
plots <- cowplot::plot_grid(gl_atac_gl_proms, neurons_atac_neurons_proms)
ggsave('examples/Germline_Neurons_Vmat-comparison.pdf', height = 6, width = 12, plots)
```

### 3.2 Normalized Vplots

Ideally, one would take into account the background ATAC-seq fragments to better estimate the molecular context in each condition. Samples might have an background fragment size higher than others and this could affect the appearance of un-normalized Vplots.

```
Vmat_background <- computeVmat(
    bam_list[['Neurons']], 
    shuffleGRanges(proms_list[['Neurons_proms']])
)
Vmat <- bam_list[['Neurons']] %>% 
    computeVmat(proms_list[['Neurons_proms']]) %>%
    normalizeVmat(
        background = Vmat_background, 
        scale = TRUE, 
        normFun = 'pctsum', 
        roll = 3
    ) %>%
    plotVmat(
        main = 'Neurons-spe. ATAC fragments over Neurons-spe. proms'
    )
ggsave('examples/Vmat_Neurons-over-neurons-REs_NORMALIZED.pdf')
# An easier way to include such background normalization is to use the 
# argument `estimate_background = TRUE`
# Scales can also be adjusted using the `breaks` argument.
neurons_atac_neurons_proms_NORM <- plotVmat(
    bam_list[['Neurons']], 
    proms_list[['Neurons_proms']], 
    estimate_background = TRUE,
    breaks = seq(0, 12, length.out = 61), 
    main = 'Neurons-spe. ATAC fragments over Neurons-spe. proms (Norm.)'
)
gl_atac_gl_proms_NORM <- plotVmat(
    bam_list[['Germline']], 
    proms_list[['Germline_proms']], 
    estimate_background = TRUE,
    breaks = seq(0, 12, length.out = 61), 
    main = 'Germline-spe. ATAC fragments over Germline-spe. proms (Norm.)'
)
plots <- cowplot::plot_grid(gl_atac_gl_proms_NORM, neurons_atac_neurons_proms_NORM)
ggsave('examples/Germline_Neurons_NORMALIZED-Vmat-comparison.pdf', height = 6, width = 12, plots)
```

To compute many different Vplots simultaneously, one can pass the two main arguments (bam\_granges and granges) to the plotVmat function using a named list.

```
list_params <- list(
    "Germline ATAC-seq over Germline proms." = list(bam_list[['Germline']], proms_list[['Germline_proms']]),
    "Germline ATAC-seq over Ubiq. proms." = list(bam_list[['Germline']], proms_list[['Ubiq._proms']]),
    "Neurons ATAC-seq over Neurons proms." = list(bam_list[['Neurons']], proms_list[['Neurons_proms']]),
    "Neurons ATAC-seq over Ubiq. proms." = list(bam_list[['Neurons']], proms_list[['Ubiq._proms']]),
    "Muscle ATAC-seq over Muscle proms." = list(bam_list[['Muscle']], proms_list[['Muscle_proms']]),
    "Muscle ATAC-seq over Ubiq. proms." = list(bam_list[['Muscle']], proms_list[['Ubiq._proms']]),
    "Hypod. ATAC-seq over Hypod. proms." = list(bam_list[['Hypod.']], proms_list[['Hypod._proms']]),
    "Hypod. ATAC-seq over Ubiq. proms." = list(bam_list[['Hypod.']], proms_list[['Ubiq._proms']]),
    "Intest. ATAC-seq over Intest. proms." = list(bam_list[['Intest.']], proms_list[['Intest._proms']]),
    "Intest. ATAC-seq over Ubiq. proms." = list(bam_list[['Intest.']], proms_list[['Ubiq._proms']])
)
plots <- plotVmat(
    list_params, 
    xlim = c(-200, 200), 
    estimate_background = TRUE, 
    cores = length(list_params)
)
p <- plots + 
    facet_wrap(~Cond., nrow = 2, dir = 'v') + 
    theme(legend.position = 'bottom') +
    theme(panel.spacing = unit(1, "lines"))
ggsave('examples/Comparison_tissue-specific-normalized-Vmats.pdf', height = 7.2, width = 18)
```

## 4 Real case uses

### 4.1. Real case use #1: Compare nucleosome patterns over tissue-specific promoters or enhancers

Let’s have a look at tissue-specific classes of promoters and the associated Vplots, in a “programmatic” approach. We already presented the possibility to pass a list of arguments to plotVmat (see section 2.2). This allows a person to plot multiple Vplots at once. Another way is to rely on parallelized computations of individual Vmats. Such simpler approach generating a list of plots allows the user to personalize each plot individually.

```
plotlist <- parallel::mclapply(seq_along(proms_list), function(K) {
    plot.proms <- parallel::mclapply(seq_along(bam_list), function(B) {
        message(names(bam_list)[B])
        plotVmat(
            bam_list[[B]], 
            proms_list[[K]],
            estimate_background = TRUE,
            main = paste0(names(bam_list)[B], '-spe. ATAC-seq over ', names(proms_list)[K], ' proms'), 
            breaks = seq(0, 12, length.out = 61), 
            ylim = c(55, 300)
        )
    }, mc.cores = length(bam_list))
    return(plot.proms)
}, mc.cores = length(proms_list))
plots <- purrr::flatten(plotlist)
p <- ggpubr::ggarrange(
    plotlist = plots, 
    nrow = 6, ncol = 6, 
    common.legend = TRUE,
    legend = "bottom"
)
ggsave('examples/Comparison-tissue-specific-normalized-Vmats_all-combinations.pdf', height = 45, width = 40)
```

It is striking that Germline promoters are characterized by prominent flanking nucleosomes, while somatic promoters are not. Ubiquitous promoters, on the other hand, are flanked by nucleosomes in all tissues.

### 4.2. Real case use #2: Nucleosome enrichment estimation

We can estimate nucleosome enrichment using the `nucleosomeEnrichment()` function. This function is used to quantify the local enrichment of a nucleosome at a certain distance from the midpoint of the genomic ranges of interest.

```
list_scores <- parallel::mclapply(
    c("Germline", "Neurons", "Muscle", "Hypod.", "Intest."), 
    function(TISSUE) {
        message('>> ', TISSUE)
        nucenrich_tissue_spe_proms <- nucleosomeEnrichment(
            bam_granges = bam_list[[TISSUE]], 
            granges = proms_list[[paste0(TISSUE, '_proms')]], 
            estimate_background = TRUE, 
            verbose = TRUE
        )
        nucenrich_ubiq_proms <- nucleosomeEnrichment(
            bam_granges = bam_list[[TISSUE]], 
            granges = proms_list[['Ubiq._proms']], 
            estimate_background = TRUE, 
            verbose = TRUE
        )
        return(list(
            'tissue-spe-proms' = nucenrich_tissue_spe_proms, 
            'ubiq-proms' = nucenrich_ubiq_proms
        ))
    }, 
    mc.cores = 5
) %>% setNames(c("Germline", "Neurons", "Muscle", "Hypod.", "Intest."))
nucenrich_scores <- data.frame(
    tissue = factor(rep(names(list_scores), each = 2), levels = names(list_scores)), 
    promoters = c(rbind(paste0(names(list_scores), ' promoters'), rep('Ubiq. promoters', 5))), 
    promoters2 = factor(rep(c(0.5, 0.8), 5)),
    score = unlist(lapply(list_scores, function(Vmat) c(Vmat[[1]]$fisher_test$estimate, Vmat[[2]]$fisher_test$estimate))), 
    is_ubiq = factor(rep(c('Over tissue-specific promoters', 'Over ubiquitous promoters'), 5))
)
p <- ggplot(nucenrich_scores, aes(
    x = tissue, 
    y = score, 
    fill = tissue, 
    group = is_ubiq
)) + 
    geom_col() + 
    facet_wrap(~is_ubiq, nrow = 2) + 
    coord_flip() + 
    labs(title = 'Flanking nucleosome enrichment score', y = 'Enrichment score', x = 'Tissue-specific data') + 
    theme_bw() + 
    theme(legend.position = 'none')
ggsave('examples/Comparison_tissue-specific-nucleosome-enrichment.pdf', height = 5, width = 5)
```

This confirms what the Vplots suggest: flanking nucleosomes are well enriched at ubiquitous promoters in all tissues. Among tissue-specific promoters, only germline promoters have a significant enrichment of flanking nucleosome.

### 4 .3. Real case use #3: Use MNase-seq data

We can also use MNase data (only this data comes from mixed tissues) to look at nucleosome patterns.

```
# Read the bam files from local folder: 
### mnase_files <- list.files(
###     '~/20181127_PE-mnase-mapping/_bam-files', 
###     pattern = 'rep-1.*q10.bam', 
###     full.names = TRUE
### )
### MNase_mixed <- importPEBamFiles(
###     mnase_files, 
###     where = GenomicRanges::reduce(GenomicRanges::resize(ce_proms, width = 2000, fix = 'center')), 
###     shift_ATAC_fragments = FALSE
### ) %>% 
###     setNames(mnase_files %>% basename() %>% gsub('.map_pe\\^rm_chrM\\^rm_blacklist\\^q10.bam', '', .)) %>% 
###     '['(names(.) %>% gsub('YA_mnase_rep-1_|U', '', .) %>% as.numeric() %>% order())
# Or simply: 
MNase_mixed <- readRDS(
    url(
        'http://ahringerlab.com/VplotR/MNase_PE_fragments.rds'
    )
)
# Then plot Vmats
list_params <- lapply(seq_along(MNase_mixed), function(B) {
    lapply(seq_along(proms_list), function(K) {
        list(
            MNase_mixed[[B]], 
            resize(proms_list[[K]], 2000, fix = 'center')
        )
    }) %>% setNames(paste0('MNase (', gsub('YA_mnase_rep-1_', '', names(MNase_mixed))[B], ') coverage \nover ', names(proms_list), ' TSSs'))
}) %>% purrr::flatten()
plots <- plotVmat(
    list_params, 
    XAXIS.CUTOFF = 2000, 
    xlim = c(-1000, 1000), 
    ylim = c(75, 200),
    breaks = seq(0, 15, length.out = 60), 
    cores = length(list_params)
)
p <- plots + 
    facet_wrap(~Cond., ncol = length(proms_list), dir = 'h') + 
    theme(legend.position = 'bottom') +
    theme(panel.spacing = unit(1, "lines")) + 
    coord_fixed(ratio = 7)
ggsave('examples/MNase-coverage_proms.pdf', height = 15, width = 18)
```

As expected, nucleosomal reads are mapped over flanking nucleosomes, specifically around ubiquitous and germline promoters. Arrays of nucleosomes are readily detected at these promoters.

## Contents

- 0. Set up the session
- 1. Import reads bam files and genomic loci files
- 2. Check distribution of fragment sizes over a specific set of segments
- 3. Vplots
  - 3.1 Quick Vplot
  - 3.2 Normalized Vplots
- 4 Real case uses
  - 4.1. Real case use #1: Compare nucleosome patterns over tissue-specific promoters or enhancers
  - 4.2. Real case use #2: Nucleosome enrichment estimation
  - 4 .3. Real case use #3: Use MNase-seq data

Developed by Jacques Serizay.

Site built with pkgdown 1.4.1.
