# Supplementary material for: Distinctive regulatory architectures of germline-active and somatic genes in C. elegans
Source: Genome Res. 2020 Dec;30(12):1752–65. doi: 10.1101/gr.265934.120 (PMC7706728; doi:10.1101/gr.265934.120)
Supplement: Supplemental Material [file supp_gr.265934.120_VplotR-0.4.0.tar.gz.zip › VplotR-0.4.0.tar.gz!/VplotR-0.4.0/docs/index.html]

VplotR package • VplotR
 


  


 


Toggle navigation


VplotR
0.4.0

- Get started
- Reference
- Articles
  - In-depth use

# VplotR v0.4.0

## Sequencing fragment lengths bear information

Sequencing libraries generated by MNase-seq, DNase-seq or ATAC-seq to study nucleosome arrangement are traditionally sequenced in a paired-end manner. This was originally useful to select 147-bp-long sequencing reads in MNase-seq, theoretically corresponding to nucleosomal DNA (#Valouev2008-mz, #Gaffney2012-yl). However, more recent approaches have elegantly used MNase-seq fragment lengths to elucidate transcription factors binding patterns (#Henikoff2011-uu). Furthermore, emerging techniques such as ATAC-seq generate genomic fragments not only from nucleosome-spanning DNA but also from nucleosome-depleted regions, and a multi-modal distribution of short or longer fragments is expected (#Buenrostro2013-ps).

## A visualization approach more powerful than traditional aggregate metaplots

Representation of ATAC-seq data is usually based on the aggregation of the linear ATAC-seq signal over multiple genomic loci. However, the distribution of fragments lengths can also give valuable information on the local arrangement of nucleosomes flanking an nucleosome-depleted region (#Schep2015-jk). Thus, using the ATAC-seq fragment lengths as a second parameter to investigate the accessibility patterns at specific genomic loci may bring insights than the traditional aggregated metaplots. This can be done by generating ATAC-seq fragment density plots.

Fragment density plots, also known as “V-plots” have been introduced for the first time by the Henikoff lab in 2011, using MNase-seq data. More recently, ATAC-seq V-plots have proven to be very instructive to understand the molecular organization of the chromatin. For instance, the nucleoATAC package relies on cross-correlation of ATAC-seq fragment density plots to accurately map nucleosome occupancy along the genome.

## Focus of VplotR

VplotR package aims to streamline the process of generating V-plots. It contains functions to import paired-end sequencing bam files and generate V-plots around genomic loci of interest. VplotR is built on ggplot2 and makes full use of its potential. As such, it is easy to generate V-plots in batches and combine them with other plots to make publication-ready figures.

The current release of VplotR is v0.4.0. This is the release which has been used in Serizay et al., submitted.

To read more about VplotR, check out the Get started vignette.

## Links

- Contact the author at at   
  https://​js2264.github.io/​
- Browse source code at at   
  https://​github.com/​js2264/​VplotR/​
- Read publication at at   
  https://​github.com/​js2264/​VplotR/​blob/​master/​vignettes/​Introduction.md

## License

- GNU v3

## Developers

- Jacques Serizay   
   Maintainer

Developed by Jacques Serizay.

Site built with pkgdown 1.4.1.
