# Supplementary material for: Distinctive regulatory architectures of germline-active and somatic genes in C. elegans
Source: Genome Res. 2020 Dec;30(12):1752–65. doi: 10.1101/gr.265934.120 (PMC7706728; doi:10.1101/gr.265934.120)
Supplement: Supplemental Material [file supp_gr.265934.120_VplotR-0.4.0.tar.gz.zip › VplotR-0.4.0.tar.gz!/VplotR-0.4.0/docs/reference/alignToTSS.html]

A function to re-align GRanges to their TSS — alignToTSS • VplotR


Toggle navigation


VplotR
0.4.0

- Get started
- Reference
- Articles
  - In-depth use

# A function to re-align GRanges to their TSS

`alignToTSS.Rd`

A function to re-align GRanges to their TSS

```
alignToTSS(granges, upstream, downstream)
```

## Arguments

|  |  |
| --- | --- |
| granges | A GRanges object with a TSS column or TSS.rev and TSS.fwd columns |
| upstream | How many bases upstream of the TSS? |
| downstream | How many bases downstream of the TSS? |

## Value

GRanges aligned to the TSS column or to TSS.rev and TSS.fwd columns

## Contents

- Arguments
- Value

Developed by Jacques Serizay.

Site built with pkgdown 1.4.1.
