# Supplementary material for: Distinctive regulatory architectures of germline-active and somatic genes in C. elegans
Source: Genome Res. 2020 Dec;30(12):1752–65. doi: 10.1101/gr.265934.120 (PMC7706728; doi:10.1101/gr.265934.120)
Supplement: Supplemental Material [file supp_gr.265934.120_VplotR-0.4.0.tar.gz.zip › VplotR-0.4.0.tar.gz!/VplotR-0.4.0/VplotR_0.3.0.tar.gz!/VplotR/inst/doc/VplotR.html]

Vignette Title


# Vignette Title

#### Vignette Author

#### 2020-02-09

Vignettes are long form documentation commonly included in packages. Because they are part of the distribution of the package, they need to be as compact as possible. The `html_vignette` output type provides a custom style sheet (and tweaks some options) to ensure that the resulting html is as small as possible. The `html_vignette` format:

- Never uses retina figures
- Has a smaller default figure size
- Uses a custom CSS stylesheet instead of the default Twitter Bootstrap style

## Vignette Info

Note the various macros within the `vignette` section of the metadata block above. These are required in order to instruct R how to build the vignette. Note that you should change the `title` field and the `\VignetteIndexEntry` to match the title of your vignette.

## Styles

The `html_vignette` template includes a basic CSS theme. To override this theme you can specify your own CSS in the document metadata as follows:

```
output: 
  rmarkdown::html_vignette:
    css: mystyles.css
```

## Figures

The figure sizes have been customised so that you can easily put two images side-by-side.

```
plot(1:10)
plot(10:1)
```

You can enable figure captions by `fig_caption: yes` in YAML:

```
output:
  rmarkdown::html_vignette:
    fig_caption: yes
```

Then you can use the chunk option `fig.cap = "Your figure caption."` in **knitr**.

## More Examples

You can write math expressions, e.g. \(Y = X\beta + \epsilon\), footnotes^1^, and tables, e.g. using `knitr::kable()`.

|  | mpg | cyl | disp | hp | drat | wt | qsec | vs | am | gear | carb |
| --- | --- | --- | --- | --- | --- | --- | --- | --- | --- | --- | --- |
| Mazda RX4 | 21.0 | 6 | 160.0 | 110 | 3.90 | 2.620 | 16.46 | 0 | 1 | 4 | 4 |
| Mazda RX4 Wag | 21.0 | 6 | 160.0 | 110 | 3.90 | 2.875 | 17.02 | 0 | 1 | 4 | 4 |
| Datsun 710 | 22.8 | 4 | 108.0 | 93 | 3.85 | 2.320 | 18.61 | 1 | 1 | 4 | 1 |
| Hornet 4 Drive | 21.4 | 6 | 258.0 | 110 | 3.08 | 3.215 | 19.44 | 1 | 0 | 3 | 1 |
| Hornet Sportabout | 18.7 | 8 | 360.0 | 175 | 3.15 | 3.440 | 17.02 | 0 | 0 | 3 | 2 |
| Valiant | 18.1 | 6 | 225.0 | 105 | 2.76 | 3.460 | 20.22 | 1 | 0 | 3 | 1 |
| Duster 360 | 14.3 | 8 | 360.0 | 245 | 3.21 | 3.570 | 15.84 | 0 | 0 | 3 | 4 |
| Merc 240D | 24.4 | 4 | 146.7 | 62 | 3.69 | 3.190 | 20.00 | 1 | 0 | 4 | 2 |
| Merc 230 | 22.8 | 4 | 140.8 | 95 | 3.92 | 3.150 | 22.90 | 1 | 0 | 4 | 2 |
| Merc 280 | 19.2 | 6 | 167.6 | 123 | 3.92 | 3.440 | 18.30 | 1 | 0 | 4 | 4 |

Also a quote using `>`:

> “He who gives up [code] safety for [code] speed deserves neither.” (via)

---

1. A footnote here.↩
