# Supplementary material for: Distinctive regulatory architectures of germline-active and somatic genes in C. elegans
Source: Genome Res. 2020 Dec;30(12):1752–65. doi: 10.1101/gr.265934.120 (PMC7706728; doi:10.1101/gr.265934.120)
Supplement: Supplemental Material [file supp_gr.265934.120_VplotR-0.4.0.tar.gz.zip › VplotR-0.4.0.tar.gz!/VplotR-0.4.0/docs/reference/computeNucleosomeEnrichmentOverBackground.html]

A function to compute nucleosome enrichment of a Vmat. Should only be used
internally. — computeNucleosomeEnrichmentOverBackground • VplotR


Toggle navigation


VplotR
0.4.0

- Get started
- Reference
- Articles
  - In-depth use

# A function to compute nucleosome enrichment of a Vmat. Should only be used internally.

`computeNucleosomeEnrichmentOverBackground.Rd`

A function to compute nucleosome enrichment of a Vmat. Should only be used
internally.

```
computeNucleosomeEnrichmentOverBackground(Vmat, background = NULL,
  plus1_nuc_only = FALSE, minus1_nuc = list(c(xmin = -150, xmax = -50),
  c(ymin = 165, ymax = 270)), minus1_nuc_neg = list(c(xmin = -150, xmax =
  -50), c(ymin = 50, ymax = 145)), plus1_nuc = list(c(xmin = 60, xmax =
  160), c(ymin = 165, ymax = 270)), plus1_nuc_neg = list(c(xmin = 60,
  xmax = 160), c(ymin = 50, ymax = 145)), ...)
```

## Arguments

|  |  |
| --- | --- |
| Vmat | A Vmat computed by nucleosomeEnrichment function |
| background | a background Vmat |
| plus1\_nuc\_only | Should compute nucleosome enrichment only for +1 nucleosome? |
| minus1\_nuc | list where the -1 nucleosome is located |
| minus1\_nuc\_neg | where the background of the -1 nucleosome is located |
| plus1\_nuc | where the +1 nucleosome is located |
| plus1\_nuc | where the background of the +1 nucleosome is located |

## Value

list

## Contents

- Arguments
- Value

Developed by Jacques Serizay.

Site built with pkgdown 1.4.1.
