# Supplementary material for: Distinctive regulatory architectures of germline-active and somatic genes in C. elegans
Source: Genome Res. 2020 Dec;30(12):1752–65. doi: 10.1101/gr.265934.120 (PMC7706728; doi:10.1101/gr.265934.120)
Supplement: Supplemental Material [file supp_gr.265934.120_VplotR-0.4.0.tar.gz.zip › VplotR-0.4.0.tar.gz!/VplotR-0.4.0/docs/reference/computeVmat.html]

A function to compute Vmat. — computeVmat • VplotR


Toggle navigation


VplotR
0.4.0

- Get started
- Reference
- Articles
  - In-depth use

# A function to compute Vmat.

`computeVmat.Rd`

A function to compute Vmat.

```
computeVmat(reads.granges, target.granges, XAXIS.CUTOFF = 1000,
  YAXIS.CUTOFF = 1000, stranded = TRUE)
```

## Arguments

|  |  |
| --- | --- |
| reads.granges | GRanges. The paired-end fragments |
| target.granges | GRanges. The regions to map the fragments onto |
| XAXIS.CUTOFF | single integer. The x limits (-/+) of the computed Vmat |
| YAXIS.CUTOFF | single integer. The y upper limit of the computed Vmat |
| stranded | Boolean. Should the Vmat be stranded? If TRUE, negative-strand features are flipped. |

## Value

A table object

## Contents

- Arguments
- Value

Developed by Jacques Serizay.

Site built with pkgdown 1.4.1.
