# Supplementary material for: Distinctive regulatory architectures of germline-active and somatic genes in C. elegans
Source: Genome Res. 2020 Dec;30(12):1752–65. doi: 10.1101/gr.265934.120 (PMC7706728; doi:10.1101/gr.265934.120)
Supplement: Supplemental Material [file supp_gr.265934.120_VplotR-0.4.0.tar.gz.zip › VplotR-0.4.0.tar.gz!/VplotR-0.4.0/docs/reference/getChromSizes.html]

A function to get / estimate the size of chromosomes for the input genome — getChromSizes • VplotR


Toggle navigation


VplotR
0.4.0

- Get started
- Reference
- Articles
  - In-depth use

# A function to get / estimate the size of chromosomes for the input genome

`getChromSizes.Rd`

A function to get / estimate the size of chromosomes for the input genome

```
getChromSizes(x, ...)
```

## Value

GRanges of whole chromosomes for the input genome

## Contents

- Value

Developed by Jacques Serizay.

Site built with pkgdown 1.4.1.
