# Supplementary material for: Distinctive regulatory architectures of germline-active and somatic genes in C. elegans
Source: Genome Res. 2020 Dec;30(12):1752–65. doi: 10.1101/gr.265934.120 (PMC7706728; doi:10.1101/gr.265934.120)
Supplement: Supplemental Material [file supp_gr.265934.120_VplotR-0.4.0.tar.gz.zip › VplotR-0.4.0.tar.gz!/VplotR-0.4.0/docs/reference/getFragmentsDistribution.html]

A function to compute sizes distribution for PE fragments — getFragmentsDistribution • VplotR


Toggle navigation


VplotR
0.4.0

- Get started
- Reference
- Articles
  - In-depth use

# A function to compute sizes distribution for PE fragments

`getFragmentsDistribution.Rd`

A function to compute sizes distribution for PE fragments

```
getFragmentsDistribution(fragments, granges_list = NULL,
  extend_granges = c(-500, 500), limits = c(0, 600), roll = 3)
```

## Arguments

|  |  |
| --- | --- |
| fragments | GRanges object containing PE fragments. See importPEBamFiles for more details. |
| extend\_granges | A numeric vector of length 2. How the GRanges should be extended. |
| limits | A numeric vector of length 2. Only consider fragments within this window of sizes. |
| roll | Integer Apply a moving average of this size |
| granges | GRanges. cam be a list of different sets of GRanges. should overlap in order to be considered when estimating fragment sizes. |

## Value

A list of tbl, one for each .bam file.

## Contents

- Arguments
- Value

Developed by Jacques Serizay.

Site built with pkgdown 1.4.1.
