# Supplementary material for: Distinctive regulatory architectures of germline-active and somatic genes in C. elegans
Source: Genome Res. 2020 Dec;30(12):1752–65. doi: 10.1101/gr.265934.120 (PMC7706728; doi:10.1101/gr.265934.120)
Supplement: Supplemental Material [file supp_gr.265934.120_VplotR-0.4.0.tar.gz.zip › VplotR-0.4.0.tar.gz!/VplotR-0.4.0/docs/reference/importPEBamFiles.html]

A function to import paired end bam files as GRanges. Can be quite lengthy
for .bam files with 5+ millions fragments. — importPEBamFiles • VplotR


Toggle navigation


VplotR
0.4.0

- Get started
- Reference
- Articles
  - In-depth use

# A function to import paired end bam files as GRanges. Can be quite lengthy for .bam files with 5+ millions fragments.

`importPEBamFiles.Rd`

A function to import paired end bam files as GRanges. Can be quite lengthy
for .bam files with 5+ millions fragments.

```
importPEBamFiles(files, where = NULL, max_insert_size = 1000,
  shift_ATAC_fragments = FALSE, verbose = TRUE)
```

## Arguments

|  |  |
| --- | --- |
| files | A character vector. Each element of the vector is the path of an individual .bam file. |
| where | GRanges. Only import the fragments mapping to the input GRanges (can fasten the import process a lot). (mapping to the same coordinates) be removed? |
| max\_insert\_size | Integer Filter out fragments larger than this size. |
| shift\_ATAC\_fragments | If the fragments come from ATAC-seq, one might want to shift the extremities by +5 / -4 bp. |
| verbose | Boolean |

## Value

A GRanges object containing fragments from the input .bam file.

## Contents

- Arguments
- Value

Developed by Jacques Serizay.

Site built with pkgdown 1.4.1.
