# Supplementary material for: Distinctive regulatory architectures of germline-active and somatic genes in C. elegans
Source: Genome Res. 2020 Dec;30(12):1752–65. doi: 10.1101/gr.265934.120 (PMC7706728; doi:10.1101/gr.265934.120)
Supplement: Supplemental Material [file supp_gr.265934.120_VplotR-0.4.0.tar.gz.zip › VplotR-0.4.0.tar.gz!/VplotR-0.4.0/docs/reference/index.html]

Function reference • VplotR


Toggle navigation


VplotR
0.4.0

- Get started
- Reference
- Articles
  - In-depth use

# Reference

| Plotting fragment density graphs | |
| --- | --- |
| `plotVmat()` | A function to plot a Vmat |
| `computeVmat()` | A function to compute Vmat. |
| `scaleVmat()` | A function to scale a Vmat. |
| `normalizeVmat()` | A function to normalized a Vmat to a given background. |
| `shuffleVmat()` | A function to shuffle a Vmat |
| Computing flanking nucleosome enrichment scores | |
| --- | --- |
| `nucleosomeEnrichment()` | A function to compute nucleosome enrichment over a set of GRanges |
| `computeNucleosomeEnrichmentOverBackground()` | A function to compute nucleosome enrichment of a Vmat. Should only be used internally. |
| Helpers | |
| --- | --- |
| `importPEBamFiles()` | A function to import paired end bam files as GRanges. Can be quite lengthy for .bam files with 5+ millions fragments. |
| `shiftATACGranges()` | A function to shift GRanges fragments by 5/-4. This is useful when dealing with fragments coming from ATAC-seq. |
| `getFragmentsDistribution()` | A function to compute sizes distribution for PE fragments |

## Contents

- Plotting fragment density graphs
- Computing flanking nucleosome enrichment scores
- Helpers

Developed by Jacques Serizay.

Site built with pkgdown 1.4.1.
