# Supplementary material for: Distinctive regulatory architectures of germline-active and somatic genes in C. elegans
Source: Genome Res. 2020 Dec;30(12):1752–65. doi: 10.1101/gr.265934.120 (PMC7706728; doi:10.1101/gr.265934.120)
Supplement: Supplemental Material [file supp_gr.265934.120_VplotR-0.4.0.tar.gz.zip › VplotR-0.4.0.tar.gz!/VplotR-0.4.0/docs/reference/nucleosomeEnrichment.GRanges.html]

A function to compute nucleosome enrichment over a set of GRanges — nucleosomeEnrichment.GRanges • VplotR


Toggle navigation


VplotR
0.4.0

- Get started
- Reference
- Articles
  - In-depth use

# A function to compute nucleosome enrichment over a set of GRanges

`nucleosomeEnrichment.GRanges.Rd`

A function to compute nucleosome enrichment over a set of GRanges

```
# S3 method for GRanges
nucleosomeEnrichment(bam_granges, granges,
  normalize = TRUE, Vmt2 = NULL, estimate_background = TRUE,
  plus1_nuc_only = FALSE, verbose = TRUE, genome = "ce11",
  roll = 1, normFun = "pctsum", ...)
```

## Arguments

|  |  |
| --- | --- |
| bam\_granges | GRanges. The paired-end fragments |
| granges | GRanges. The regions to map the fragments onto |
| estimate\_background | Boolean Should the background be estimated automatically? |
| plus1\_nuc\_only | Should compute nucleosome enrichment only for +1 nucleosome? |
| verbose | Boolean |
| genome | a BSgenome object. See getChromSizes for more details |

## Value

list

## Contents

- Arguments
- Value

Developed by Jacques Serizay.

Site built with pkgdown 1.4.1.
