# Supplementary material for: Distinctive regulatory architectures of germline-active and somatic genes in C. elegans
Source: Genome Res. 2020 Dec;30(12):1752–65. doi: 10.1101/gr.265934.120 (PMC7706728; doi:10.1101/gr.265934.120)
Supplement: Supplemental Material [file supp_gr.265934.120_VplotR-0.4.0.tar.gz.zip › VplotR-0.4.0.tar.gz!/VplotR-0.4.0/docs/reference/plotVmat.GRanges.html]

A function to plot a computed Vmat — plotVmat.GRanges • VplotR


Toggle navigation


VplotR
0.4.0

- Get started
- Reference
- Articles
  - In-depth use

# A function to plot a computed Vmat

`plotVmat.GRanges.Rd`

A function to plot a computed Vmat

```
# S3 method for GRanges
plotVmat(bam_granges, granges, XAXIS.CUTOFF = 1000,
  YAXIS.CUTOFF = 1000, stranded = TRUE, normalize = TRUE,
  Vmat2 = NULL, estimate_background = TRUE,
  background_granges = NULL, normFun = "pctsum", roll = 3,
  return_Vmat = FALSE, genome = "ce11", ...)
```

## Arguments

|  |  |
| --- | --- |
| bam\_granges | GRanges. The paired-end fragments |
| granges | GRanges. The regions to map the fragments onto |
| XAXIS.CUTOFF | single integer. The x limits (-/+) of the computed Vmat |
| YAXIS.CUTOFF | single integer. The y upper limit of the computed Vmat |
| stranded | Boolean. Should the Vmat be stranded? If TRUE, negative-strand features are flipped |
| normalize | Boolean Should the Vmat be normalized? |
| Vmat2 | table A Vmat pre-computed over a background |
| estimate\_background | Boolean Should the background be estimated automatically? |
| background\_granges | GRanges The genomic loci used to compute the background Vmat |
| normFun | string A Vmat can be scaled relative to its median ('pctmedian'), to its mean ('pctmean'), to its max ('pctmax'). Otherwise it could be zscore-d entirely ('Zscore') or by rows ('rowZscore') or by columns ('colZscore'). |
| roll | integer to use as the window to smooth the Vmat rows by rolling mean |
| return\_Vmat | Boolean. Should the function return the computed Vmat rather than the plot? |
| genome | a BSgenome object. See getChromSizes for more details |

## Value

A Vmat ggplot

## Contents

- Arguments
- Value

Developed by Jacques Serizay.

Site built with pkgdown 1.4.1.
