# Supplementary material for: Distinctive regulatory architectures of germline-active and somatic genes in C. elegans
Source: Genome Res. 2020 Dec;30(12):1752–65. doi: 10.1101/gr.265934.120 (PMC7706728; doi:10.1101/gr.265934.120)
Supplement: Supplemental Material [file supp_gr.265934.120_VplotR-0.4.0.tar.gz.zip › VplotR-0.4.0.tar.gz!/VplotR-0.4.0/docs/reference/plotVmat.default.html]

A function to plot a computed Vmat — plotVmat.default • VplotR


Toggle navigation


VplotR
0.4.0

- Get started
- Reference
- Articles
  - In-depth use

# A function to plot a computed Vmat

`plotVmat.default.Rd`

A function to plot a computed Vmat

```
# S3 method for default
plotVmat(Vmat, pdf = NULL, HM.COLOR.CUTOFF = 90,
  colors = c(colorRampPalette(rev(RColorBrewer::brewer.pal("Spectral", n
  = 10))[1:5])(30),
  colorRampPalette(rev(RColorBrewer::brewer.pal("Spectral", n =
  10))[6:10])(30)), breaks = NULL, xlim = c(-250, 250), ylim = c(50,
  300), main = "", xlab = "Distance from center of elements",
  ylab = "Fragment length", ...)
```

## Arguments

|  |  |
| --- | --- |
| Vmat | A computed Vmat (should be normalized) |
| pdf | string save the plot in a pdf |
| HM.COLOR.CUTOFF | Integer should be between 0 and 100. Used to automatically scale the range of colors (ebst to keep between 90 and 100) |
| colors | a vector of colors |
| breaks | a vector of breaks. length(breaks) == length(colors) + 1 |
| xlim | vector of two integers x limits |
| ylim | vector of two integers y limits |
| main | string Title of the plot |
| xlab | string x-axis label |
| ylab | string y-axis label |

## Value

A Vmat ggplot

## Contents

- Arguments
- Value

Developed by Jacques Serizay.

Site built with pkgdown 1.4.1.
