# Supplementary material for: Distinctive regulatory architectures of germline-active and somatic genes in C. elegans
Source: Genome Res. 2020 Dec;30(12):1752–65. doi: 10.1101/gr.265934.120 (PMC7706728; doi:10.1101/gr.265934.120)
Supplement: Supplemental Material [file supp_gr.265934.120_VplotR-0.4.0.tar.gz.zip › VplotR-0.4.0.tar.gz!/VplotR-0.4.0/docs/reference/scaleVmat.html]

A function to scale a Vmat. — scaleVmat • VplotR


Toggle navigation


VplotR
0.4.0

- Get started
- Reference
- Articles
  - In-depth use

# A function to scale a Vmat.

`scaleVmat.Rd`

A function to scale a Vmat.

```
scaleVmat(Vmat, FUN = "pctsum")
```

## Arguments

|  |  |
| --- | --- |
| Vmat |  |
| FUN | string. A Vmat can be scaled relative to its median ('pctmedian'), to its mean ('pctmean'), to its max ('pctmax'). Otherwise it could be zscore-d entirely ('Zscore') or by rows ('rowZscore') or by columns ('colZscore'). |

## Value

A table object

## Contents

- Arguments
- Value

Developed by Jacques Serizay.

Site built with pkgdown 1.4.1.
