# Supplementary material for: Distinctive regulatory architectures of germline-active and somatic genes in C. elegans
Source: Genome Res. 2020 Dec;30(12):1752–65. doi: 10.1101/gr.265934.120 (PMC7706728; doi:10.1101/gr.265934.120)
Supplement: Supplemental Material [file supp_gr.265934.120_VplotR-0.4.0.tar.gz.zip › VplotR-0.4.0.tar.gz!/VplotR-0.4.0/docs/reference/shiftATACGranges.html]

A function to shift GRanges fragments by 5/-4. This is useful when dealing
with fragments coming from ATAC-seq. — shiftATACGranges • VplotR


Toggle navigation


VplotR
0.4.0

- Get started
- Reference
- Articles
  - In-depth use

# A function to shift GRanges fragments by 5/-4. This is useful when dealing with fragments coming from ATAC-seq.

`shiftATACGranges.Rd`

A function to shift GRanges fragments by 5/-4. This is useful when dealing
with fragments coming from ATAC-seq.

```
shiftATACGranges(g, pos_shift = 5, neg_shift = 4)
```

## Arguments

|  |  |
| --- | --- |
| g | GRanges of ATAC-seq fragments |
| pos\_shift | Integer. How many bases should fragments on direct strand be shifted by? |
| neg\_shift | Integer. How many bases should fragments on negative strand be shifted by? |

## Value

A GRanges object containing fragments from the input .bam file.

## Contents

- Arguments
- Value

Developed by Jacques Serizay.

Site built with pkgdown 1.4.1.
