# Supplementary material for: Distinctive regulatory architectures of germline-active and somatic genes in C. elegans
Source: Genome Res. 2020 Dec;30(12):1752–65. doi: 10.1101/gr.265934.120 (PMC7706728; doi:10.1101/gr.265934.120)
Supplement: Supplemental Material [file supp_gr.265934.120_VplotR-0.4.0.tar.gz.zip › VplotR-0.4.0.tar.gz!/VplotR-0.4.0/docs/reference/shuffleGRanges.html]

A function to shuffle GRanges along a given genome
Internaly, this function relies on bedTools shuffle, so the bedTools
suite has to be installed on the workstation. — shuffleGRanges • VplotR


Toggle navigation


VplotR
0.4.0

- Get started
- Reference
- Articles
  - In-depth use

# A function to shuffle GRanges along a given genome Internaly, this function relies on bedTools shuffle, so the bedTools suite has to be installed on the workstation.

`shuffleGRanges.Rd`

A function to shuffle GRanges along a given genome
Internaly, this function relies on bedTools shuffle, so the bedTools
suite has to be installed on the workstation.

```
shuffleGRanges(granges, genome = NULL,
  opt.shuffle = "-chrom -noOverlapping", exclude_itself = TRUE,
  SEED = 222)
```

## Arguments

|  |  |
| --- | --- |
| granges | A GRanges object to shuffle |
| genome | a BSgenome object. See getChromSizes for more details |
| opt.shuffle | string of options (in single quotes) to pass to bedtools shuffle |
| exclude\_itself | Boolean. Should the shuffled GRanges overlap the input GRanges or not? |
| SEED | Integer. Make shuffling reproducible |

## Value

a GRanges object with shuffled ranges

## Contents

- Arguments
- Value

Developed by Jacques Serizay.

Site built with pkgdown 1.4.1.
