# Supplementary material for: Distinctive regulatory architectures of germline-active and somatic genes in C. elegans
Source: Genome Res. 2020 Dec;30(12):1752–65. doi: 10.1101/gr.265934.120 (PMC7706728; doi:10.1101/gr.265934.120)
Supplement: Supplemental Material [file supp_gr.265934.120_periodicDNA-0.2.0.tar.gz.zip › periodicDNA-0.2.0.tar.gz!/periodicDNA-0.2.0/docs/404.html]

Page not found (404) • periodicDNA


Toggle navigation


periodicDNA
0.2.0

- Reference

# Page not found (404)

Content not found. Please use links in the navbar.

Developed by Jacques Serizay.

Site built with pkgdown 1.4.1.
