# Supplementary material for: Distinctive regulatory architectures of germline-active and somatic genes in C. elegans
Source: Genome Res. 2020 Dec;30(12):1752–65. doi: 10.1101/gr.265934.120 (PMC7706728; doi:10.1101/gr.265934.120)
Supplement: Supplemental Material [file supp_gr.265934.120_periodicDNA-0.2.0.tar.gz.zip › periodicDNA-0.2.0.tar.gz!/periodicDNA-0.2.0/docs/index.html]

periodicDNA package • periodicDNA
 


  


 


Toggle navigation


periodicDNA
0.2.0

- Reference

# periodicDNA

## Introduction

This R package helps the user to identify very short sequences (e.g. di- or tri-nucleotides) present periodically in a set of genomic loci (typically regulatory elements). It is not aimed at identifying motifs separated by a conserved distance; for this type of analysis, please visit MEME website.

## Installation

periodicDNA can be installed from Github as follows:

```
install.packages("devtools")
devtools::install_github("js2264/periodicDNA")
library(periodicDNA)
```

## Overview

To begin, a genome sequence and a set of genomic loci must be defined. Let’s focus on the C. elegans genome for now, and more specifically around its TSSs.

```
require(magrittr)
require(GenomicRanges)
require(ggplot2)
ce_seq <- Biostrings::getSeq(BSgenome.Celegans.UCSC.ce11::BSgenome.Celegans.UCSC.ce11)
ce_proms <- readRDS(url('http://ahringerlab.com/VplotR/promoters_Granges.rds'))
```

## Dinucleotide periodicity over a set of Genomic Ranges

Let’s look at the TT 10-bp periodicity strength around ubiquitous promoters:

```
MOTIF <- 'TT'
ubiq_TT <- getPeriodicity(
    alignToTSS(ce_proms[ce_proms$which.tissues == 'Ubiq.'], 30, 500), 
    genome = ce_seq, 
    motif = MOTIF, 
    cores = 4
)
list_plots <- plotPeriodicityResults(ubiq_TT)
```

## Make track of periodicity over a set of Genomic Ranges

The other major use of this package is to generate specific tracks over a set of loci, e.g. the strength of WW 10-pb periodicity over promoters.  
**Important note:** We recommand to run this command across at least a dozen of processors (use the `PROCS` argument). This command will take several hours and possibly up to a day to run. It typically takes one day to produce a periodicity track over 15,000 GRanges of 150 bp (with default parameters) using `PROCS = 12`. We highly recommand the user to run this command in a new `screen` session.

```
generatePeriodicityTrack(
    ce_seq,
    granges = ce_proms, 
    MOTIF = 'TT',
    FREQ = 1/10,
    PROCS = 12, 
    GENOME.WINDOW.SIZE = 100, 
    GENOME.WINDOW.SLIDING = 2, # can be 1 for single-base resolution
    BIN.WINDOW.SIZE = 60, # Set BIN.WINDOW.SIZE == GENOME.WINDOW.SIZE for no sliding window
    BIN.WINDOW.SLIDING = 5, 
    bw.file = 'TT-10-bp-periodicity_over-proms_gwin100_bwin60_bslide5.bw'
)
```

## Other functions of the package

Please read the Introduction vignette for a full presentation of the package functions.

## Links

- Contact the author at at   
  https://​js2264.github.io/​
- Browse source code at at   
  https://​github.com/​js2264/​periodicDNA/​
- Read publication at at   
  https://​github.com/​js2264/​periodicDNA/​blob/​master/​vignettes/​Introduction.md

## License

- GPL-3

## Developers

- Jacques Serizay   
   Maintainer

Developed by Jacques Serizay.

Site built with pkgdown 1.4.1.
