# Supplementary material for: Distinctive regulatory architectures of germline-active and somatic genes in C. elegans
Source: Genome Res. 2020 Dec;30(12):1752–65. doi: 10.1101/gr.265934.120 (PMC7706728; doi:10.1101/gr.265934.120)
Supplement: Supplemental Material [file supp_gr.265934.120_periodicDNA-0.2.0.tar.gz.zip › periodicDNA-0.2.0.tar.gz!/periodicDNA-0.2.0/docs/reference/alignToTSS.html]

A function to re-align a GRanges object to a provided set of coordinates
(either the TSS column or the TSS.fwd and TSS.rev columns) — alignToTSS • periodicDNA


Toggle navigation


periodicDNA
0.2.0

- Reference

# A function to re-align a GRanges object to a provided set of coordinates (either the TSS column or the TSS.fwd and TSS.rev columns)

`alignToTSS.Rd`

A function to re-align a GRanges object to a provided set of coordinates
(either the TSS column or the TSS.fwd and TSS.rev columns)

```
alignToTSS(granges, upstream = 0, downstream = 1)
```

## Arguments

|  |  |
| --- | --- |
| granges | A stranded GRanges object with a TSS column or TSS.rev and TSS.fwd columns |
| upstream | How many bases upstream of the TSS should the GRanges object by extended by? [Default: 0] |
| downstream | How many bases downstream of the TSS should the GRanges object by extended by? [Default: 1] |

## Value

GRanges aligned to the TSS column or to TSS.rev and TSS.fwd columns,
and extended by upstream/downstream bp.

## Contents

- Arguments
- Value

Developed by Jacques Serizay.

Site built with pkgdown 1.4.1.
