# Supplementary material for: Distinctive regulatory architectures of germline-active and somatic genes in C. elegans
Source: Genome Res. 2020 Dec;30(12):1752–65. doi: 10.1101/gr.265934.120 (PMC7706728; doi:10.1101/gr.265934.120)
Supplement: Supplemental Material [file supp_gr.265934.120_periodicDNA-0.2.0.tar.gz.zip › periodicDNA-0.2.0.tar.gz!/periodicDNA-0.2.0/docs/reference/chunkWrapper.html]

Internal function — chunkWrapper • periodicDNA


Toggle navigation


periodicDNA
0.2.0

- Reference

# Internal function

`chunkWrapper.Rd`

Internal function

```
chunkWrapper(chunk, genome, MOTIF, GENOME.WINDOW.SLIDING, BIN.WINDOW.SIZE,
  BIN.WINDOW.SLIDING, RANGE.FOR.SPECTRUM, FREQ)
```

## Arguments

|  |  |
| --- | --- |
| chunk | A GRanges from the output list obtained with getChunks |
| genome | A DNAStringSet object. Ideally, the sequence of an entire genome, obtained for instance by running `Biostrings::getSeq(BSgenome.Celegans.UCSC.ce11::BSgenome.Celegans.UCSC.ce11)`. |
| MOTIF | String Oligonucleotide of interest. |
| GENOME.WINDOW.SLIDING | Integer The increment between bins over GRanges (default 2). |
| BIN.WINDOW.SIZE | Integer The width of the bins to split each primary bin in (default 60). |
| BIN.WINDOW.SLIDING | Integer The increment between secondary bins (default 5). |
| RANGE.FOR.SPECTRUM | Numeric vector The distances between nucleotides to take into consideration when performing Fast Fourier Transform (default 1:50). |
| FREQ | Float The frequence of the dinucleotide to study (default 1/10). |

## Value

GRanges a GRanges object with a score column containing estimated
periodicity

## Contents

- Arguments
- Value

Developed by Jacques Serizay.

Site built with pkgdown 1.4.1.
