# Supplementary material for: Distinctive regulatory architectures of germline-active and somatic genes in C. elegans
Source: Genome Res. 2020 Dec;30(12):1752–65. doi: 10.1101/gr.265934.120 (PMC7706728; doi:10.1101/gr.265934.120)
Supplement: Supplemental Material [file supp_gr.265934.120_periodicDNA-0.2.0.tar.gz.zip › periodicDNA-0.2.0.tar.gz!/periodicDNA-0.2.0/docs/reference/generatePeriodicityTrack.html]

Core function to generate a track of periodicity strenght of a given
dinucleotide at a given frequency, over a set of chosen GRanges. — generatePeriodicityTrack • periodicDNA


Toggle navigation


periodicDNA
0.2.0

- Reference

# Core function to generate a track of periodicity strenght of a given dinucleotide at a given frequency, over a set of chosen GRanges.

`generatePeriodicityTrack.Rd`

Core function to generate a track of periodicity strenght of a given
dinucleotide at a given frequency, over a set of chosen GRanges.

```
generatePeriodicityTrack(genome = Biostrings::getSeq(BSgenome.Celegans.UCSC.ce11::BSgenome.Celegans.UCSC.ce11),
  granges, MOTIF = "WW", EXTEND.GRANGES = 1000,
  GENOME.WINDOW.SIZE = 100, GENOME.WINDOW.SLIDING = 2,
  BIN.WINDOW.SIZE = 60, BIN.WINDOW.SLIDING = 5,
  RANGE.FOR.SPECTRUM = 1:50, FREQ = 1/10, PROCS = 12,
  bw.file = NULL)
```

## Arguments

|  |  |
| --- | --- |
| genome | DNAStringSet object. Ideally, the sequence of an entire genome, obtained for instance by running `Biostrings::getSeq(BSgenome.Celegans.UCSC.ce11::BSgenome.Celegans.UCSC.ce11)`. |
| granges | GRanges object (with seqnames overlapping the names of genome). |
| MOTIF | String Oligonucleotide of interest. |
| EXTEND.GRANGES | Integer The width the GRanges are going to be extended to (default 1000). |
| GENOME.WINDOW.SIZE | Integer The width of the bins to split the GRanges objects in (default 100). |
| GENOME.WINDOW.SLIDING | Integer The increment between bins over GRanges (default 2). |
| BIN.WINDOW.SIZE | Integer The width of the bins to split each primary bin in (default 60). |
| BIN.WINDOW.SLIDING | Integer The increment between secondary bins (default 5). |
| RANGE.FOR.SPECTRUM | Numeric vector The distances between nucleotides to take into consideration when performing Fast Fourier Transform (default 1:50). |
| FREQ | Float The frequence of the dinucleotide to study (default 1/10). |
| PROCS | Integer Split the workload over several processors (default 12). |
| bw.file | String. The name of the output bigWig track |

## Value

NULL A bigWig track in the working directory.

## Contents

- Arguments
- Value

Developed by Jacques Serizay.

Site built with pkgdown 1.4.1.
