# Supplementary material for: Distinctive regulatory architectures of germline-active and somatic genes in C. elegans
Source: Genome Res. 2020 Dec;30(12):1752–65. doi: 10.1101/gr.265934.120 (PMC7706728; doi:10.1101/gr.265934.120)
Supplement: Supplemental Material [file supp_gr.265934.120_periodicDNA-0.2.0.tar.gz.zip › periodicDNA-0.2.0.tar.gz!/periodicDNA-0.2.0/docs/reference/getCovMatrix.html]

Internal function — getCovMatrix • periodicDNA


Toggle navigation


periodicDNA
0.2.0

- Reference

# Internal function

`getCovMatrix.Rd`

Internal function

```
getCovMatrix(g, bw, norm = "none", verbose = FALSE)
```

## Arguments

|  |  |
| --- | --- |
| g | GRanges to map a track onto |
| bw | RleList a track from rtraklayer::import |
| norm | character Should the signal be normalized ('none', 'zscore' or 'log2')? |
| verbose | Boolean Should the function be verbose? |

## Value

A square numerical matrix of signal values over the GRanges

## Contents

- Arguments
- Value

Developed by Jacques Serizay.

Site built with pkgdown 1.4.1.
