# Supplementary material for: Distinctive regulatory architectures of germline-active and somatic genes in C. elegans
Source: Genome Res. 2020 Dec;30(12):1752–65. doi: 10.1101/gr.265934.120 (PMC7706728; doi:10.1101/gr.265934.120)
Supplement: Supplemental Material [file supp_gr.265934.120_periodicDNA-0.2.0.tar.gz.zip › periodicDNA-0.2.0.tar.gz!/periodicDNA-0.2.0/docs/reference/getPeriodicity.DNAStringSet.html]

Function to compute the overall periodicity of a motif over a set of
sequences. — getPeriodicity.DNAStringSet • periodicDNA


Toggle navigation


periodicDNA
0.2.0

- Reference

# Function to compute the overall periodicity of a motif over a set of sequences.

`getPeriodicity.DNAStringSet.Rd`

```
# S3 method for DNAStringSet
getPeriodicity(seqs, bg_seqs = NULL,
  model_background = FALSE, motif = "WW", RANGE_FOR_SPECTRUM = 1:200,
  period = seq(2, 20, 1), plot = FALSE, cores = 2, roll = 3,
  verbose = TRUE, sample = 0, doZscore = TRUE)
```

## Arguments

|  |  |
| --- | --- |
| seqs | a DNAStringSet |
| bg\_seqs | a DNAStringSet (ideally from random loci) for genome background model |
| model\_background | Boolean Should the genome background be taken into account during normalization? |
| motif | a dinucleotide of interest |
| RANGE\_FOR\_SPECTRUM | Numeric vector The distances between nucleotides to take into consideration when performing Fast Fourier Transform. |
| period | Vector a numerical vector of periods to extract. |
| plot | Boolean Should the FFT results be plotted? |
| cores | Integer How many processors should be used to parallelize the mapping? |
| roll | Integer Window to smooth the distribution of pairwise distances (default: 3, to discard the 3-bp periodicity of dinucleotides which can be very strong in vertebrate genomes) |
| verbose | Boolean |
| sample | Integer if > 0, will randomly sample this many integers from the dists vector before normalization. This ensures consistency when looking at periodicity in different genomes, since different genomes will have different GC |

## Contents

- Arguments

Developed by Jacques Serizay.

Site built with pkgdown 1.4.1.
