# Supplementary material for: Distinctive regulatory architectures of germline-active and somatic genes in C. elegans
Source: Genome Res. 2020 Dec;30(12):1752–65. doi: 10.1101/gr.265934.120 (PMC7706728; doi:10.1101/gr.265934.120)
Supplement: Supplemental Material [file supp_gr.265934.120_periodicDNA-0.2.0.tar.gz.zip › periodicDNA-0.2.0.tar.gz!/periodicDNA-0.2.0/docs/reference/getPeriodicity.GRanges.html]

Core function — getPeriodicity.GRanges • periodicDNA


Toggle navigation


periodicDNA
0.2.0

- Reference

# Core function

`getPeriodicity.GRanges.Rd`

Core function

```
# S3 method for GRanges
getPeriodicity(granges, bg = NULL,
  genome = Biostrings::getSeq(BSgenome.Celegans.UCSC.ce11::BSgenome.Celegans.UCSC.ce11),
  ...)
```

## Arguments

|  |  |
| --- | --- |
| granges | a GRanges |
| bg | a GRanges to estimate background periodicity. |
| genome | DNAStringSet object. The sequence of an entire genome, obtained for instance by running `Biostrings::getSeq(BSgenome.Celegans.UCSC.ce11::BSgenome.Celegans.UCSC.ce11)`. |
| ... | other parameters forwarded to getPeriodicity.DNAStringSet() |

## Value

List a list containing the results of getPeriodicity function. The
dists vector is the raw vector of all pairwise distances between dinucleotides.
The hist dataframe is the distribution of distances over RANGE\_FOR\_SPECTRUM.
The normalized\_hist is the raw histogram normalized for
decay over increasing distances between pairs of dinucleotides.
The spectra object is the output of the FFT applied over normalized\_hist.
The PSD dataframe contains power spectrum density scores over given
frequencies.
The signal\_to\_noise\_ratio is a dataframe containing enrichment scores of
dinucleotide periodicity, for each period in the period vector.
The motif character is the analysed dinucleotide.

## Contents

- Arguments
- Value

Developed by Jacques Serizay.

Site built with pkgdown 1.4.1.
