# Supplementary material for: Distinctive regulatory architectures of germline-active and somatic genes in C. elegans
Source: Genome Res. 2020 Dec;30(12):1752–65. doi: 10.1101/gr.265934.120 (PMC7706728; doi:10.1101/gr.265934.120)
Supplement: Supplemental Material [file supp_gr.265934.120_periodicDNA-0.2.0.tar.gz.zip › periodicDNA-0.2.0.tar.gz!/periodicDNA-0.2.0/docs/reference/getPeriodicity.html]

Function to compute the overall periodicity of a motif over sequence(s). — getPeriodicity • periodicDNA


Toggle navigation


periodicDNA
0.2.0

- Reference

# Function to compute the overall periodicity of a motif over sequence(s).

`getPeriodicity.Rd`

Function to compute the overall periodicity of a motif over sequence(s).

```
getPeriodicity(x, ...)
```

## Value

List a list containing the results of getPeriodicity function. The
dists vector is the raw vector of all distances between any possible dinucleotide.
The hist data.frame is the distribution of distances over RANGE\_FOR\_SPECTRUM.
The normalized\_hist is the raw hist, normalized for decay over increasing distances.
The spectra object is the output of the FFT applied over normalized\_hist.
The PSD data frame is the power spectrum density scores over given frequencies.
The signal\_to\_noise\_ratio is a data.frame containing enrichment scores of TT
periodicity, for the periods in the period vector. The motif object is the
dinucleotide being analysed.

## Contents

- Value

Developed by Jacques Serizay.

Site built with pkgdown 1.4.1.
