# Supplementary material for: Distinctive regulatory architectures of germline-active and somatic genes in C. elegans
Source: Genome Res. 2020 Dec;30(12):1752–65. doi: 10.1101/gr.265934.120 (PMC7706728; doi:10.1101/gr.265934.120)
Supplement: Supplemental Material [file supp_gr.265934.120_periodicDNA-0.2.0.tar.gz.zip › periodicDNA-0.2.0.tar.gz!/periodicDNA-0.2.0/docs/reference/index.html]

Function reference • periodicDNA


Toggle navigation


periodicDNA
0.2.0

- Reference

# Reference

| All functions | |
| --- | --- |
| `alignToTSS()` | A function to re-align a GRanges object to a provided set of coordinates (either the TSS column or the TSS.fwd and TSS.rev columns) |
| `binWrapper()` | Internal function |
| `chunkWrapper()` | Internal function |
| `cleanUpDirectory()` | Internal function |
| `deconvolveBidirectionalPromoters()` | A function to duplicate bi-directional GRanges into + and - stranded GRanges. |
| `generatePeriodicityTrack()` | Core function to generate a track of periodicity strenght of a given dinucleotide at a given frequency, over a set of chosen GRanges. |
| `getChunks()` | Internal function |
| `getCovMatrix()` | Internal function |
| `getPeriodicity(<DNAStringSet>)` | Function to compute the overall periodicity of a motif over a set of sequences. |
| `getPeriodicity(<GRanges>)` | Core function |
| `getPeriodicity()` | Function to compute the overall periodicity of a motif over sequence(s). |
| `na.remove()` | A tidyverse-compatible function to remove NAs from a vector |
| `na.replace()` | A tidyverse-compatible function to replace NAs in a vector by a given value |
| `namedListToLongFormat()` | A function to easily coerce a named list into a long data.frame |
| `normalizeHistogram()` | Internal function to normalize a pairwise distance |
| `partitionBin()` | Internal function |
| `partitionGenome()` | Internal function |
| `plotAggregateCoverage(<CompressedRleList>)` | A function to plot aggregated signals (from a CompressedRleList) over sets of GRanges |
| `plotAggregateCoverage()` | A function to plot aggregated signals over sets of GRanges |
| `plotAggregateCoverage(<SimpleRleList>)` | A function to plot aggregated signals (from a SimpleRleList) over sets of GRanges |
| `plotAggregateCoverage(<list>)` | A function to plot aggregated signals (from a named list of signal tracks) over sets of GRanges |
| `plotPeriodicityResults()` | Plotting function |
| `scaleBigWigs()` | A function to z-score a bigWig track imported by rtracklayer. |
| `unlistResults()` | Internal function |
| `withSeq()` | Tidyverse-compatible function to add sequence to an existing GRanges |

## Contents

- All functions

Developed by Jacques Serizay.

Site built with pkgdown 1.4.1.
