# Supplementary material for: Distinctive regulatory architectures of germline-active and somatic genes in C. elegans
Source: Genome Res. 2020 Dec;30(12):1752–65. doi: 10.1101/gr.265934.120 (PMC7706728; doi:10.1101/gr.265934.120)
Supplement: Supplemental Material [file supp_gr.265934.120_periodicDNA-0.2.0.tar.gz.zip › periodicDNA-0.2.0.tar.gz!/periodicDNA-0.2.0/docs/reference/na.replace.html]

A tidyverse-compatible function to replace NAs in a vector by a given value — na.replace • periodicDNA


Toggle navigation


periodicDNA
0.2.0

- Reference

# A tidyverse-compatible function to replace NAs in a vector by a given value

`na.replace.Rd`

A tidyverse-compatible function to replace NAs in a vector by a given value

```
na.replace(x, value)
```

## Arguments

|  |  |
| --- | --- |
| x | vector |
| value | Replace NAs by this variable |

## Value

A vector with NA replaced by value

## Contents

- Arguments
- Value

Developed by Jacques Serizay.

Site built with pkgdown 1.4.1.
