# Supplementary material for: Distinctive regulatory architectures of germline-active and somatic genes in C. elegans
Source: Genome Res. 2020 Dec;30(12):1752–65. doi: 10.1101/gr.265934.120 (PMC7706728; doi:10.1101/gr.265934.120)
Supplement: Supplemental Material [file supp_gr.265934.120_periodicDNA-0.2.0.tar.gz.zip › periodicDNA-0.2.0.tar.gz!/periodicDNA-0.2.0/docs/reference/partitionBin.html]

Internal function — partitionBin • periodicDNA


Toggle navigation


periodicDNA
0.2.0

- Reference

# Internal function

`partitionBin.Rd`

Internal function

```
partitionBin(grange, genome, BIN.WINDOW.SIZE, BIN.WINDOW.SLIDING)
```

## Arguments

|  |  |
| --- | --- |
| grange | A GRanges object of length 1 |
| genome | A DNAStringSet object. Ideally, the sequence of an entire genome, obtained for instance by running `Biostrings::getSeq(BSgenome.Celegans.UCSC.ce11::BSgenome.Celegans.UCSC.ce11)`. |
| BIN.WINDOW.SIZE | Integer The width of the bins to split each primary bin in (default 60). |
| BIN.WINDOW.SLIDING | Integer The increment between secondary bins (default 5). |

## Value

list A GRanges object

## Contents

- Arguments
- Value

Developed by Jacques Serizay.

Site built with pkgdown 1.4.1.
