# Supplementary material for: Distinctive regulatory architectures of germline-active and somatic genes in C. elegans
Source: Genome Res. 2020 Dec;30(12):1752–65. doi: 10.1101/gr.265934.120 (PMC7706728; doi:10.1101/gr.265934.120)
Supplement: Supplemental Material [file supp_gr.265934.120_periodicDNA-0.2.0.tar.gz.zip › periodicDNA-0.2.0.tar.gz!/periodicDNA-0.2.0/docs/reference/partitionGenome.html]

Internal function — partitionGenome • periodicDNA


Toggle navigation


periodicDNA
0.2.0

- Reference

# Internal function

`partitionGenome.Rd`

Internal function

```
partitionGenome(genome, granges.extended_large, granges.extended,
  GENOME.WINDOW.SIZE, GENOME.WINDOW.SLIDING)
```

## Arguments

|  |  |
| --- | --- |
| genome | A DNAStringSet object. Ideally, the sequence of an entire genome, obtained for instance by running `Biostrings::getSeq(BSgenome.Celegans.UCSC.ce11::BSgenome.Celegans.UCSC.ce11)`. |
| granges.extended\_large | A GRanges object. |
| granges.extended | A GRanges object. `Biostrings::getSeq(BSgenome.Celegans.UCSC.ce11::BSgenome.Celegans.UCSC.ce11)`. |
| GENOME.WINDOW.SIZE | An integer. The width of the bins to split the GRanges objects in (default 100). |
| GENOME.WINDOW.SLIDING | An integer. The increment between bins over GRanges (default 2). |

## Value

granges.partionned A Granges object corresponding to the binned genome.

## Contents

- Arguments
- Value

Developed by Jacques Serizay.

Site built with pkgdown 1.4.1.
