# Supplementary material for: Distinctive regulatory architectures of germline-active and somatic genes in C. elegans
Source: Genome Res. 2020 Dec;30(12):1752–65. doi: 10.1101/gr.265934.120 (PMC7706728; doi:10.1101/gr.265934.120)
Supplement: Supplemental Material [file supp_gr.265934.120_periodicDNA-0.2.0.tar.gz.zip › periodicDNA-0.2.0.tar.gz!/periodicDNA-0.2.0/docs/reference/plotAggregateCoverage.CompressedRleList.html]

A function to plot aggregated signals (from a CompressedRleList)
over sets of GRanges — plotAggregateCoverage.CompressedRleList • periodicDNA


Toggle navigation


periodicDNA
0.2.0

- Reference

# A function to plot aggregated signals (from a CompressedRleList) over sets of GRanges

`plotAggregateCoverage.CompressedRleList.Rd`

A function to plot aggregated signals (from a CompressedRleList)
over sets of GRanges

```
# S3 method for CompressedRleList
plotAggregateCoverage(bw, granges, ...)
```

## Arguments

|  |  |
| --- | --- |
| bw | a single signal track (CompressedRleList class) |
| granges | a GRanges object or a list of GRanges |

## Value

A plot of aggregated signals

## Contents

- Arguments
- Value

Developed by Jacques Serizay.

Site built with pkgdown 1.4.1.
