# Supplementary material for: Distinctive regulatory architectures of germline-active and somatic genes in C. elegans
Source: Genome Res. 2020 Dec;30(12):1752–65. doi: 10.1101/gr.265934.120 (PMC7706728; doi:10.1101/gr.265934.120)
Supplement: Supplemental Material [file supp_gr.265934.120_periodicDNA-0.2.0.tar.gz.zip › periodicDNA-0.2.0.tar.gz!/periodicDNA-0.2.0/docs/reference/plotAggregateCoverage.SimpleRleList.html]

A function to plot aggregated signals (from a SimpleRleList)
over sets of GRanges — plotAggregateCoverage.SimpleRleList • periodicDNA


Toggle navigation


periodicDNA
0.2.0

- Reference

# A function to plot aggregated signals (from a SimpleRleList) over sets of GRanges

`plotAggregateCoverage.SimpleRleList.Rd`

A function to plot aggregated signals (from a SimpleRleList)
over sets of GRanges

```
# S3 method for SimpleRleList
plotAggregateCoverage(bw, granges,
  colors = rep(c("#991919", "#1232D9", "#3B9B46", "#D99B12", "#7e7e7e",
  "#D912D4", "#9E7FCC", "#B0E797", "#D1B3B3", "#23A4A3", "#000000"), 5),
  xlab = "Center of elements", ylab = "Score", xlim = NULL,
  ylim = NULL, quartiles = c(0.025, 0.975), verbose = FALSE,
  bin = 1, plot_central = TRUE, runInParallel = TRUE,
  split_by_granges = FALSE, norm = "none", ...)
```

## Arguments

|  |  |
| --- | --- |
| bw | a single signal track (SimpleRleList class) |
| granges | a GRanges object or a list of GRanges |
| colors | a vector of colors |
| xlab | x axis label |
| ylab | y axis label |
| xlim | y axis limits |
| ylim | y axis limits |
| quartiles | Which quantiles to use to determine y scale automatically? |
| verbose | Boolean |
| bin | Integer Width of the window to use to smooth values by zoo::rollMean |
| plot\_central | Boolean Draw a vertical line at 0 |
| runInParallel | Boolean Should the plots be computed in parallel using mclapply? |
| split\_by\_granges | Boolean Facet plots over the sets of GRanges |
| norm | character Should the signal be normalized ('none', 'zscore' or 'log2')? |

## Value

A plot of aggregated signals

## Contents

- Arguments
- Value

Developed by Jacques Serizay.

Site built with pkgdown 1.4.1.
