# Supplementary material for: Distinctive regulatory architectures of germline-active and somatic genes in C. elegans
Source: Genome Res. 2020 Dec;30(12):1752–65. doi: 10.1101/gr.265934.120 (PMC7706728; doi:10.1101/gr.265934.120)
Supplement: Supplemental Material [file supp_gr.265934.120_periodicDNA-0.2.0.tar.gz.zip › periodicDNA-0.2.0.tar.gz!/periodicDNA-0.2.0/docs/reference/plotAggregateCoverage.html]

A function to plot aggregated signals over sets of GRanges — plotAggregateCoverage • periodicDNA


Toggle navigation


periodicDNA
0.2.0

- Reference

# A function to plot aggregated signals over sets of GRanges

`plotAggregateCoverage.Rd`

A function to plot aggregated signals over sets of GRanges

```
plotAggregateCoverage(x, ...)
```

## Value

A plot of aggregated signals

## Contents

- Value

Developed by Jacques Serizay.

Site built with pkgdown 1.4.1.
