# Supplementary material for: Distinctive regulatory architectures of germline-active and somatic genes in C. elegans
Source: Genome Res. 2020 Dec;30(12):1752–65. doi: 10.1101/gr.265934.120 (PMC7706728; doi:10.1101/gr.265934.120)
Supplement: Supplemental Material [file supp_gr.265934.120_periodicDNA-0.2.0.tar.gz.zip › periodicDNA-0.2.0.tar.gz!/periodicDNA-0.2.0/docs/reference/plotPeriodicityResults.html]

Plotting function — plotPeriodicityResults • periodicDNA


Toggle navigation


periodicDNA
0.2.0

- Reference

# Plotting function

`plotPeriodicityResults.Rd`

Plotting function

```
plotPeriodicityResults(results, HIST_YLIM = c(-1.5, 1.5))
```

## Arguments

|  |  |
| --- | --- |
| results | The output of getPeriodicity function. |
| HIST\_YLIM | Vector a numerical vector of length 2, to specify the y-axis limits of the first plot (normalized distribution of pairwise distances). |

## Value

list A list containing three ggplots

## Contents

- Arguments
- Value

Developed by Jacques Serizay.

Site built with pkgdown 1.4.1.
