# Supplementary material for: Distinctive regulatory architectures of germline-active and somatic genes in C. elegans
Source: Genome Res. 2020 Dec;30(12):1752–65. doi: 10.1101/gr.265934.120 (PMC7706728; doi:10.1101/gr.265934.120)
Supplement: Supplemental Material [file supp_gr.265934.120_periodicDNA-0.2.0.tar.gz.zip › periodicDNA-0.2.0.tar.gz!/periodicDNA-0.2.0/docs/reference/withSeq.html]

Tidyverse-compatible function to add sequence to an existing GRanges — withSeq • periodicDNA


Toggle navigation


periodicDNA
0.2.0

- Reference

# Tidyverse-compatible function to add sequence to an existing GRanges

`withSeq.Rd`

Tidyverse-compatible function to add sequence to an existing GRanges

```
withSeq(granges, genome)
```

## Arguments

|  |  |
| --- | --- |
| granges | A GRanges object |
| genome | DNAStringSet object. Ideally, the sequence of an entire genome, obtained for instance by running |

## Value

GRanges with a seq column containing the sequence of the GRanges.

## Contents

- Arguments
- Value

Developed by Jacques Serizay.

Site built with pkgdown 1.4.1.
